# Genome-wide identification and expression analysis of calcium-dependent protein kinase and its closely related kinase genes in *Capsicum annuum*

**DOI:** 10.3389/fpls.2015.00737

**Published:** 2015-09-15

**Authors:** Hanyang Cai, Junbin Cheng, Yan Yan, Zhuoli Xiao, Jiazhi Li, Shaoliang Mou, Ailian Qiu, Yan Lai, Deyi Guan, Shuilin He

**Affiliations:** ^1^National Education Ministry, Key Laboratory of Plant Genetic Improvement and Comprehensive Utilization, Fujian Agriculture and Forestry UniversityFuzhou, China; ^2^College of Life Science, Fujian Agriculture and Forestry UniversityFuzhou, China; ^3^College of Crop Science, Fujian Agriculture and Forestry UniversityFuzhou, China

**Keywords:** CDPKs, CRKs, *Capsicum annuum*, salt, heat, and *R. solanacearum*

## Abstract

As Ca2+ sensors and effectors, calcium-dependent protein kinases (CDPKs) play important roles in plant growth, development, and response to environmental cues. However, no CDPKs have been characterized in *Capsicum annuum* thus far. Herein, a genome wide comprehensive analysis of genes encoding CDPKs and CDPK-related protein kinases (CRKs) was performed in pepper, a total of 31 CDPK genes and five closely related kinase genes were identified, which were phylogenetically divided into four distinct subfamilies and unevenly distributed across nine chromosomes. Conserved sequence and exon-intron structures were found to be shared by pepper CDPKs within the same subfamily, and the expansion of the CDPK family in pepper was found to be due to segmental duplication events. Five CDPKs in the *C. annuum* variety *CM334* were found to be mutated in the *Chiltepin* variety, and one CDPK present in *CM334* was lost in *Chiltepin*. The majority of CDPK and CRK genes were expressed in different pepper tissues and developmental stages, and 10, 12, and 8 CDPK genes were transcriptionally modified by salt, heat, and *Ralstonia solanacearum* stresses, respectively. Furthermore, these genes were found to respond specifically to one stress as well as respond synergistically to two stresses or three stresses, suggesting that these CDPK genes might be involved in the specific or synergistic response of pepper to salt, heat, and *R. solanacearum*. Our results lay the foundation for future functional characterization of pepper CDPK and its closely related gene families.

## Introduction

In their natural habitat, plants frequently encounter different abiotic and biotic stresses, and have evolved a complicated defense system to defend themselves against such stresses. It is crucial for plants to recognize and perceive stress in order to comprehensively reprogram their metabolism and switch to defense mode. Upon challenge with biotic or abiotic stresses, Ca^2+^ flux across the plasma membrane is generally triggered, leading to a rapid increase in the cytoplasmic Ca^2+^ concentration with varying frequency, amplitude, and duration dependent on the stress cues. These calcium concentration changes are sensed and decoded by different Ca^2+^ sensors/Ca^2+^-binding proteins, including calmodulins (CaMs), calmodulin-like proteins (CaMLs), calcineurin B-like proteins (CBLs), and calcium-dependent protein kinases (CDPKs), which subsequently result in different downstream defense responses. However, the underlying mechanism remains largely unknown.

The large family of CDPKs is characterized by an N-variable domain, a protein kinase domain, an autoinhibitory domain, and a CaM-like domain (Cheng et al., 2002). These unique features enable CDPKs to function as Ca^2+^ sensors and effectors and to play important roles in regulating the downstream components of calcium signaling. A subset of CDPK genes in this family exhibits inducible expression patterns and have been previously demonstrated to play pivotal roles not only in plant response to abiotic stresses such as drought (Saijo et al., 2000; Ludwig et al., 2004; Chen et al., 2013; Jiang et al., 2013; Vivek et al., 2013; Campo et al., 2014), cold (Saijo et al., 2000; Martín and Busconi, 2001; Ludwig et al., 2004; Chen et al., 2013), salinity (Saijo et al., 2000; Ludwig et al., 2004; Vivek et al., 2013; Campo et al., 2014), heat shock (Wan et al., 2007; Chang et al., 2009), dehydration (Ludwig et al., 2004; Wan et al., 2007), arsenic stress (Huang et al., 2012), and cadmium stress (Chmielowska-Bak et al., 2013), but also in plant response to biotic stresses such as pathogen infection (Freymark et al., 2007; Kobayashi et al., 2007; Asano et al., 2012; Fu et al., 2013), herbivore attack (Romeis and Herde, 2014), and production of reactive oxygen species (Kobayashi et al., 2007). In addition, CDPK genes have also found to be involved in plant growth and developmental processes such as fruit development (Shen et al., 2004; Jain et al., 2011), spikelet fertility (Wei et al., 2014), leaf senescence (Li et al., 2006), flowering (Jaworski et al., 2012), pollen tube growth (Estruch et al., 1994), root development (Ivashuta et al., 2005), cell division and differentiation, and cell death (Yoon et al., 1999; Lee et al., 2003). CDPK genes in rice genome exhibit differential expression levels in the stamen, panicle, endosperm, and callus (Ye et al., 2009). The signaling pathways mediated by CDPKs appear to be complicated—for example, crosstalk between the CDPK and MAPK signaling pathways has been found (Wurzinger et al., 2011; Ding et al., 2013)—and the underlying mechanism of the biological processes regulated by CDPK genes remains to be elucidated.

CDPKs are encoded by a large gene family: genome-wide analyses have identified 34 CDPK genes in *Arabidopsis* (Cheng et al., 2002), 31 in rice (Asano et al., 2005; Ray et al., 2007), 40 in maize (Kong et al., 2013), 20 in wheat (Li et al., 2008), 30 in *Populus* (Zuo et al., 2013), and 41 in cotton (Liu et al., 2014). Members of the CDPK family differ in their sequences, expression patterns, and subcellular targeting, and can be subdivided into four distinct subfamilies (Frattini et al., 1999; Dammann et al., 2003; Ray et al., 2007; Wan et al., 2007; Zuo et al., 2013); members within the same subfamily shared similar intron-exon organization (Zuo et al., 2013; Liu et al., 2014). However, diversity in the CDPK family among different plant species was also observed. For example, less than half of the *Arabidopsis* CDPK genes had *Populus* homologs. In contrast, generally two or more *Populus* homologs were found for each *Arabidopsis* CDPK gene (Zuo et al., 2013).

Pepper (*Capsicum annuum*) is a vegetable crop of great agricultural and economical importance, and is also a typical species of Solanaceae with soil-borne diseases. Heavy losses in pepper production are frequently caused by various soil-borne diseases as well as different abiotic stresses such as drought, extreme temperatures, and salinity. Understanding the molecular mechanisms underlying resistance/tolerance to diseases or stress is beneficial in engineering improved crop disease resistance or stress tolerance, and functional characterization of CDPKs is a feasible approach to dissect the these molecular mechanisms. Recent genetic evidence indicates that plant CDPK genes transcriptionally upregulated during biotic and abiotic stress responses can have important roles in these stresses resistance or tolerance(Chico et al., 2002; Asano et al., 2012; Fu et al., 2013; Jiang et al., 2013; Wei et al., 2014), for example, OsCPK9 transcription was induced by abscisic acid (ABA), PEG6000, and NaCl treatments, and OsCPK9 overexpression enhanced., while OsCPK9 RNA interference decreased drought stress tolerance(Wei et al., 2014), and the expression of OsCPK10 was strongly induced following treatment with a *Magnaporthe grisea* elicitor and rice OsCPK10 acts a crucial regulator in plant immune responses (Fu et al., 2013). To get a comprehensive understanding of CDKPs in pepper and their possible involvements in pepper's stress responses, a genome-wide identification and expression analysis of CDPKs was performed in the present study, and a total of 31 CDPK genes and 5 CRK genes were found. These CDPKs were grouped based on their phylogenetic relationships into four subgroups and were located to specific chromosomes. The transcriptional expression of CDPK genes in pepper plants against salt, heat treatment, and *Ralstonia solanacearum* inoculation was also analyzed.

## Materials and methods

### Database search for CDPK genes in pepper, phylogenetic analysis, and structural divergence

The protein sequences for 34 *Arabidopsis* CDPKs were obtained from the Arabidopsis Information Resource (http://www.Arabidopsis.org/). Sequences from the pepper genome database were downloaded from http://peppersequence.genomics.cn/page/species/index.jsp. For the identification of the pepper CDPK gene family, *Arabidopsis* CDPKs protein sequences were used to search the pepper genome and NCBI databases using BLASTP. Multiple alignments of amino acid sequences were aligned using Clustal X (Zhou et al., 2009). A phylogenetic tree was created according to the neighbor-joining method using the MEGA5.0 program (Tamura et al., 2011). The gene structures were displayed using an online tool (http://gsds.cbi.pku.edu.cn/). cDNA sequences and gene sequences were downloaded from this website: http://peppersequence.genomics.cn.

### Plant materials and growth condition

Pepper (*C. annuum)* cultivar GZ03 was provided by the pepper breeding group in Fujian Agriculture and Forestry University. The seeds of pepper GZ03 were sown in a soil mix [peat moss:perlite, 2:1 (v/v)] in plastic pots and were placed in a greenhouse at 25°C, 60–70 mmol photons m^−2^ s^−1^, a relative humidity of 70%, and a 16-h light/8-h dark photoperiod.

### Pathogens and inoculation procedures

*R. solanacearum strain* FJC100301 was isolated previously in our lab and amplified according to the method of Dang et al. (2013). The bacterial cell solution was diluted to 10^8^ cfu mL^−1^ (OD_600_ = 0.8) with 10 mM MgCl_2_. Pepper plants were inoculated by infiltrating 100 μL of the resulting *R. solanacearum* suspension into the third leaves from the apical meristem using a syringe with a needle. The leaves were harvested at the indicated time points for the preparation of total RNA.

### Salt stress and high temperature treatments

For salt stress treatment, eight-leaves pepper plants were treated with 250 mM NaCl and the plants with water as control. The salt-treated plants and the control plants were placed in a greenhouse under the same conditions mentioned above. The leaves were harvested at the indicated time points for the preparation of RNA. High temperature treatment was carried out in a growth chamber at 43°C, 100% humidity, 60–70 mmol photons m^−2^ s^−1^, and a 16-h light/8-h dark photoperiod. Normal pepper plants that were not exposed to salt stress and high temperature treatment were used as controls. The leaves were harvested at the indicated time points for the preparation of total RNA.

### Quantitative real-time PCR

To determine the relative transcript levels of selected genes, real-time PCR was performed with specific primers (Table S1 for gene-specific primers) according to the manufacturer's instructions for the Bio-Rad Real-time PCR system (Foster City, CA, USA) and the SYBR Premix Ex Taq II system (TaKaRa Perfect Real Time). Total RNA was isolated from pepper plants using Trizol reagent (Invitrogen, Carlsbad, CA, USA), and reverse-transcribed using the PrimeScript RT-PCR kit (TaKaRa, Dalian, China). A 10-fold dilution of the resulting cDNA was amplified using the SYBR Premix Ex Taq II kit and the Bio-Rad Real-time PCR system in a 10-μL volume with the following program: 95°C for 30 s; 40 cycles of 95°C for 5 s and 60°C for 34 s; 95°C for 15 s. Amplification of the target genes was monitored during each cycle by SYBR green fluorescence. The Ct (threshold cycle), defined as the real-time PCR cycle at which a statistically significant increase in reporter fluorescence was first detected, was used as a measure for the starting copy numbers of the target genes. Three replicates of each experiment were performed. Data were analyzed by the Livak method (Livak and Schmittgen, 2001) and expressed as a normalized relative expression level (2^−ΔΔCT^) of the respective genes. The relative transcript levels of the analyzed pepper genes were normalized to the transcript levels of *CaActin*. In each case, three technical replicates were performed for each of at least three independent biological replicates.

## Results

### Identification of CDPK and CDPK-related protein kinase (CRK) genes in pepper genome

The recently published genome of *C. annuum* varieties CM334 and Chiltepin make it possible to identify all pepper CDPK genes (Kim et al., 2014a). Blast searches of the pepper genome of CM334 were performed by using all of the 34 CDPK sequences in *Arabidopsis* as baits, and a total of 31 putative CDPKs and 5 CDPK-related genes were identified (Table 1, and Datasheet 1 in Supplementary Material). The deduced protein products of the 31 CDPK genes range in molecular mass from 52.7 to 69.7 kDa, and all of them contain the typical CDPK structure, including an N-variable domain, a protein kinase domain, an autoinhibitory domain, and a CaM-like domain (Harper et al., 1991; Cheng et al., 2002; Harmon, 2003). In addition, all the pepper CDPK genes were predicted to have four EF-hand motifs in the CaM-like domain, which can recognize and bind Ca^2+^ molecules (Table 1, Cheng et al., 2002; Hrabak et al., 2003). The N-terminus of a subset of CDPK proteins contain a myristoylation motif, which is believed to promote protein-membrane and protein-protein interactions (Martín and Busconi, 2000; Hrabak et al., 2003). It has been reported that proteins containing myristoylation motifs tend to localize in the plasma membrane (Hrabak et al., 2003; Martín and Busconi, 2000). Among the identified 31 pepper CDPK proteins, 11 CDPKs were predicted to contain myristoylation motifs at their N-termini.

**Table 1:** **Characteristics of CDPKs from pepper genome**.

**CaCDPK**	**CaCDPK^a^**	**CaCDPK^b^**	**ORF length**	**Amino acid**	**MW (kDa)**	**N-terminal**	**N-Myristoylation**	**EF hands**
CaCDPK1	Capang01g001337	CA01g10370	1584	527	59.6	MGNCCVKP	Yes	4
CaCDPK2	Capang01g001406	CA01g08810	1743	580	64.3	MGNTCIGP	No	4
CaCDPK3	Capang01g001476	CA01g10500	1635	544	61.3	MGNCCSRG	Yes	4
CaCDPK4	Capang01g001806	CA10g17030	1833	610	69.7	MGNNCVHA	No	4
CaCDPK5	Capang01g005134	CA07g17490	1566	521	58.5	MGICVSKS	Yes	4
CaCDPK6	Capang01g005424	CA00g76700	1707	539	63.6	MGNACRGS	No	4
CaCDPK7	Capang02g000275	CA02g03450	1569	523	59.8	MGGCFSRN	Yes	4
CaCDPK8	Capang02g002320	CA00g55450	1716	572	64.5	MGNICSAS	Yes	4
CaCDPK9	Capang03g001022	CA00g37630	1621	539	60.9	MGNCNACI	No	4
CaCDPK10	Capang03g002819	CA03g10950	1635	545	60.7	MGICLSKT	Yes	4
CaCDPK11	Capang04g000135	CA04g16340	1605	535	60.4	MGLCFSKA	Yes	4
CaCDPK12	Capang04g001808	CA04g08430	1545	515	58	MEIPNAEN	No	4
CaCDPK13	Capang04g002071	CA04g03980	1782	594	67	MGSCFSSS	Yes	4
CaCDPK14	Capang04g002230	CA04g01680	1665	555	63	MGGCFSKK	Yes	4
CaCDPK15	Capang05g000414	CA05g03200	1569	523	58.8	MTMPKSEQ	No	4
CaCDPK16	Capang06g000671	CA06g16860	1638	546	62	MKSTAIDQ	No	4
CaCDPK17	Capang06g001318	CA06g11830	1503	501	56.6	MAQVAAKK	No	4
CaCDPK18	Capang06g002308	CA10g13810	1584	527	59.2	MGNCCRSP	No	4
CaCDPK19	Capang07g000013	CA00g67180	1410	469	52.7	MDSSDSAK	No	4
CaCDPK20	Capang07g000770	CA10g17200	1461	487	53.4	MANHAEEV	No	4
CaCDPK21	Capang07g000773	CA10g17230	1728	575	63.4	MGNNCVGP	No	4
CaCDPK22	Capang08g000525	CA00g32950	1525	507	56.9	MGNNCVHE	No	4
CaCDPK23	Capang08g001300	CA00g24640	1534	510	57.2	MGTCNSTL	NO	4
CaCDPK24	Capang10g002105	CA00g13850	1593	531	60	MGNCCAVP	Yes	4
CaCDPK25	Capang11g000778	CA11g11090	1599	533	59.8	MGNCCVTP	Yes	4
CaCDPK26	Capang12g000076	CA12g21580	1503	501	56.2	MQGSTTPS	No	4
CaCDPK27	Capang12g000830	CA11g11120	1605	535	59.9	MGNYCCSR	No	4
CaCDPK28	Capang12g001828	CA12g07230	1674	558	62.5	MWGSRSPD	No	4
CaCDPK29	Capang00g003107	CA09g10260	1590	529	59.8	MGNCCRSP	No	4
CaCDPK30	Capang00g003951	CA10g09970	1551	517	57.8	MGNTCRGS	No	4
CaCDPK31		CA00g35300	1687	561	61.9	MGNTCSGP	Yes	4
CaCRK	CaCRK							
CaCRK1	Capang01g000496	CA08g12420	1764	588	65.8	MGACTSKP	Yes	0
CaCRK2	Capang02g000628	CA02g02040	1731	577	64.8	MGGCTSKP	Yes	0
CaCRK3	Capang02g002836	CA02g23060	1803	601	67.3	MGACTSKP	Yes	0
CaCRK4	Capang03g001828	CA03g19510	1800	599	67	MGQCCSKG	No	0
CaCRK5	Capang03g002325	CA03g15750	1533	511	55.9	MAVAISNS	No	0

a, b the gene accession numbers from Chiltepin and CM334. The nomenclature of Chiltepin for genes is Capang^**^g######, respectively, in which “Capang” is the short name of Capsicum annuum var. glabriusculum. “^**^” represents the number of chromosomes; “g” represents genome; “######” is the identifier for a gene that is anchored to a certain chromosome.

Additionally, five CDPK-related protein kinase genes (CRKs) were identified in the pepper genome, less than the eight CRKs identified in *Arabidopsis* (Hrabak et al., 2003). These pepper CRKs range in molecular mass from 55.9 to 67.3 kDa, and their structure is similar to that of CDPKs except for the degenerate calmodulin-like domains. Consistent with previous findings that most CRKs are modified at the N-terminus (Hrabak et al., 2003; Asano et al., 2005), all the pepper CRKs except CaCRK5 contained potential myristoylation sites at their N-termini (Table 1).

**Figure 1:**
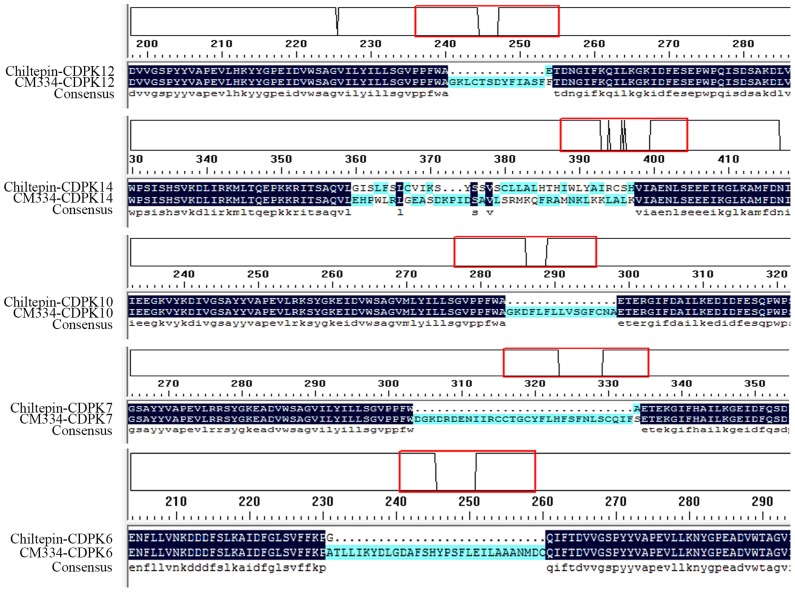
**Differences in the deduced amino acid sequences of five CDPK genes between pepper cultivars Chiltepin and CM334**. Chiltepin-CDPK: the deduced amino acid sequences of CDPK genes from Chiltepin. CM334-CDPK: the deduced amino acid sequences of CDPK genes from CM334. The red box: the detailed differences in the amino acid.

CDPKs in the pepper variety Chiltepin were also identified and CDPKs in the two pepper germplasms were compared. The sequences of the majority of the CDPK family members in the two germplasms were the same, except in the case of *CaCDPK6, CaCDPK7, CaCDPK10, CaCDPK12*, and *CaCDPK14*, in which different sequences of the same gene were found in the two germplasms (Figure 1). Moreover, *CaCDPK31*, which is present in CM334, was not found in Chiltepin.

### Phylogenetic analysis of CDPK and CRK genes and their chromosomal location

**Figure 2:**
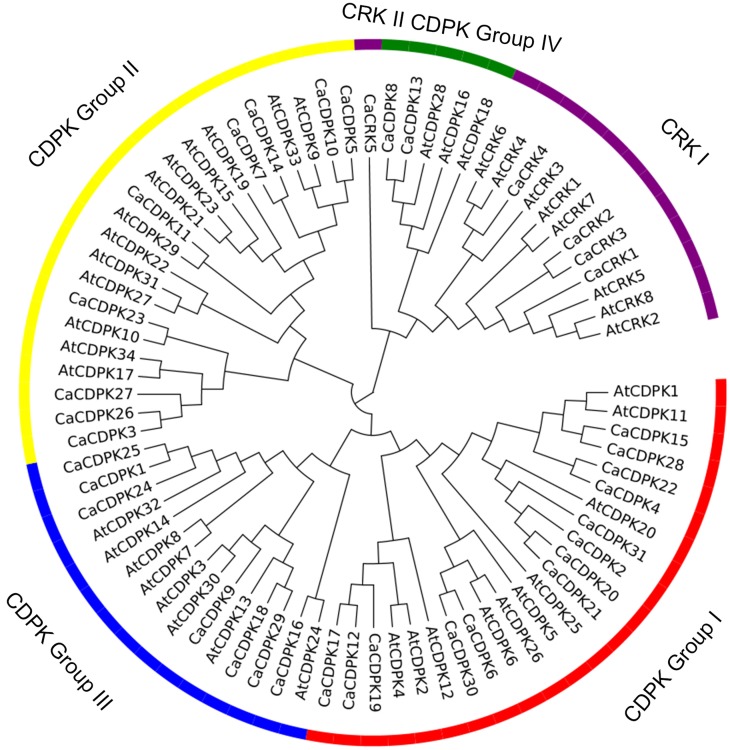
**Phylogenetic tree of CDPK and CRK genes from genomes of pepper and ***Arabidopsis*****. A neighbor-joining tree was constructed using the MEGA5.0 program with 1000 bootstrap analyses using the full length amino acid sequences of 31 pepper CDPK genes, five pepper CRK genes, 34 *Arabidopsis* CDPK genes, and eight *Arabidopsis* CRK genes. The CDPK genes were classified into four subfamilies (groups I, II, III, and IV). The CRK genes were classified into two subfamilies (CRK I and CRK II).

To examine the phylogenetic relationship among the CDPKs and CRKs in pepper, the CDPKs of *Arabidopsis* and pepper, were constructed from alignments of the full-length kinase sequences using MEGA5.0. Similar to the previous studies in *Arabidopsis*, CDPK genes in pepper were grouped into four subfamilies, with 12, eight, seven, and two members in subfamily I, II, III, and IV, respectively (Figure 2 and Table 2). Similar to the CDPKs in *Arabidopsis, Populus*, rice, and cotton, subfamilies I and II in pepper consist of large numbers of CDPKs, which exhibit a great diversity in number among different plant species: for example, four more CDPKs in subfamily I were found in cotton than in Arabidopsis, and seven more CDPKs in subfamily II were found in cotton than in poplar and rice. The members in subfamily III and IV were approximately identical across the five plant species, and subfamily IV contained the lowest number of CDPKs. Five CRKs were found in rice and pepper, whereas in *Arabidopsis* and *Populus trichocarpa*, eight and nine CRKs were found, respectively. These results indicated that differences in the number of CDPK genes in different plant species might be contributed largely by the numbers of CDPK I or CDPK II that have evolved independently among the different organisms.

**Table 2:** **The distribution of CDPKs and CRKs in ***Arabidopsis, Populus***, rice and pepper in different CDPK subfamilies**.

	***Arabidopsis thaliana***	***Populus trichocarpa***	***Oryza sativa***	***Capsicum annuum***
Group I	10	11	11	13
Group II	13	8	8	9
Group III	8	9	8	7
Group IV	3	2	2	2
CRK	8	9	5	5

**Figure 3:**
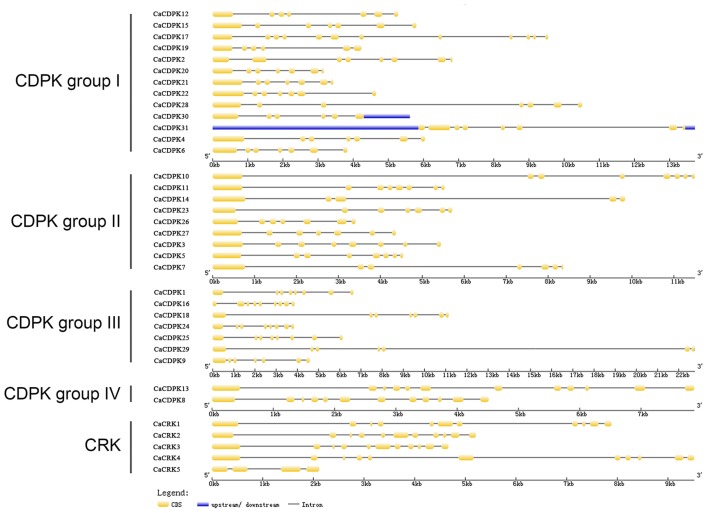
**Intron-exon organization of the 31 CDPK and five CRK genes**. The gene structures were analyzed using an online tool (http://gsds.cbi.pku.edu.cn/).

To obtain further insights into the possible structural evolution of CDPK genes in the pepper genome, diverse exon-intron organizations of CaCDPKs were compared. As shown in Figure 3 and Table 3, CaCDPKs clustered in the same subfamily shared very similar exon-intron structures. Most members of subfamily I possessed seven exons, with the exception of *CaCDPK2* and *CaCDPK17* (12 exons). Members in subfamily II harbored diverse numbers of exons: eight exons were found in *CaCDPK3, CaCDPK5, CaCDPK10*, and *CaCDPK11*, seven exons were found in *CaCDPK7, CaCDPK23, CaCDPK26*, and *CaCDPK27*, and five exons were found in *CaCDPK14*. In subfamily III, *CaCDPK16*, and *CaCDPK18* had nine exons, *CaCDPK1, CaCDPK24*, and *CaCDPK25* had eight exons, and *CaCDPK9* had seven exons. The two members in subfamily IV both had 12 exons. The results indicate that CDPK genes with higher homogenous sequences tend to have the same numbers of exons, interestingly, members of subfamily I and subfamily IV have the same numbers of exons in pepper and cotton.

**Table 3:** **Introns and exons in CDPK and CRK genes of pepper**.

**CaCDPK**	**CaCDPK**	**CaCDPK**	**Exons**	**Introns**
CaCDPK1	Capang01g001337	CA01g10370	8	7
CaCDPK2	Capang01g001406	CA01g08810	8	7
CaCDPK3	Capang01g001476	CA01g10500	8	7
CaCDPK4	Capang01g001806	CA10g17030	7	6
CaCDPK5	Capang01g005134	CA07g17490	8	7
CaCDPK6	Capang01g005424	CA00g76700	7	6
CaCDPK7	Capang02g000275	CA02g03450	7	6
CaCDPK8	Capang02g002320	CA00g55450	12	11
CaCDPK9	Capang03g001022	CA00g37630	7	6
CaCDPK10	Capang03g002819	CA03g10950	8	7
CaCDPK11	Capang04g000135	CA04g16340	8	7
CaCDPK12	Capang04g001808	CA04g08430	7	6
CaCDPK13	Capang04g002071	CA04g03980	12	11
CaCDPK14	Capang04g002230	CA04g01680	5	4
CaCDPK15	Capang05g000414	CA05g03200	7	6
CaCDPK16	Capang06g000671	CA06g16860	9	8
CaCDPK17	Capang06g001318	CA06g11830	12	11
CaCDPK18	Capang06g002308	CA10g13810	9	8
CaCDPK19	Capang07g000013	CA00g67180	6	5
CaCDPK20	Capang07g000770	CA10g17200	7	6
CaCDPK21	Capang07g000773	CA10g17230	7	6
CaCDPK22	Capang08g000525	CA00g32950	7	6
CaCDPK23	Capang08g001300	CA00g24640	7	6
CaCDPK24	Capang10g002105	CA00g13850	8	7
CaCDPK25	Capang11g000778	CA11g11090	8	7
CaCDPK26	Capang12g000076	CA12g21580	7	6
CaCDPK27	Capang12g000830	CA11g11120	7	6
CaCDPK28	Capang12g001828	CA12g07230	7	6
CaCDPK29	Capang00g003107	CA09g10260	7	6
CaCDPK30	Capang00g003951	CA10g09970	6	5
CaCDPK31		CA00g35300	8	7
CaCRK	CaCRK			
CaCRK1	Capang01g000496	CA08g12420	11	10
CaCRK2	Capang02g000628	CA02g02040	11	10
CaCRK3	Capang02g002836	CA02g23060	11	10
CaCRK4	Capang03g001828	CA03g19510	11	10
CaCRK5	Capang03g002325	CA03g15750	4	3

Among the 31 pepper *CDPKs*, 28 genes were mapped onto the chromosomes (Table 1). The largest number of CDPK genes (six) were located to chromosome 1, four CDPK genes were located to chromosome 4, and chromosomes 7 and 12 were found to harbor three CDPK genes each. Chromosome 2, 3, 8, and 12 were each found to harbor two CDPK genes. One CDPK gene was found on chromosomes 5, 10, and 11. No CDPK gene was mapped to Chromosome 9. Three genes (*CaCDPK29, CaCDPK30*, and *CaCDPK31*) could not be placed on any chromosome; these may belong to some gap regions in pseudomolecules. The five CRK genes were also mapped to different chromosomes, Chromosome 2 and 3 were found to harbor two CRK genes each, whereas one CRK gene was found on chromosome 1 (Table 1).

To elucidate the expanded mechanism of the CDPK gene family in *C. annuum*, gene duplication events, including tandem and segmental duplications, were investigated. A total of eight duplicated CDPK gene pairs, *CaCDPK1*/*CaCDPK25, CaCDPK3*/*CaCDPK26, CaCDPK4*/*CaCDPK22, CaCDPK6*/*CaCDPK30, CaCDPK8*/*CaCDPK13, CaCDPK12*/*CaCDPK17, CaCDPK18*/*CaCDPK29, and CaCDPK20*/*CaCDPK21*, and one CRK pair, *CaCRK2*/*CaCRK3*, were found in the *C. annuum* genome; all of these were segmental duplicates.

### Pepper CDPK genes and CRK genes are expressed in different tissues in pepper plants

**Figure 4:**
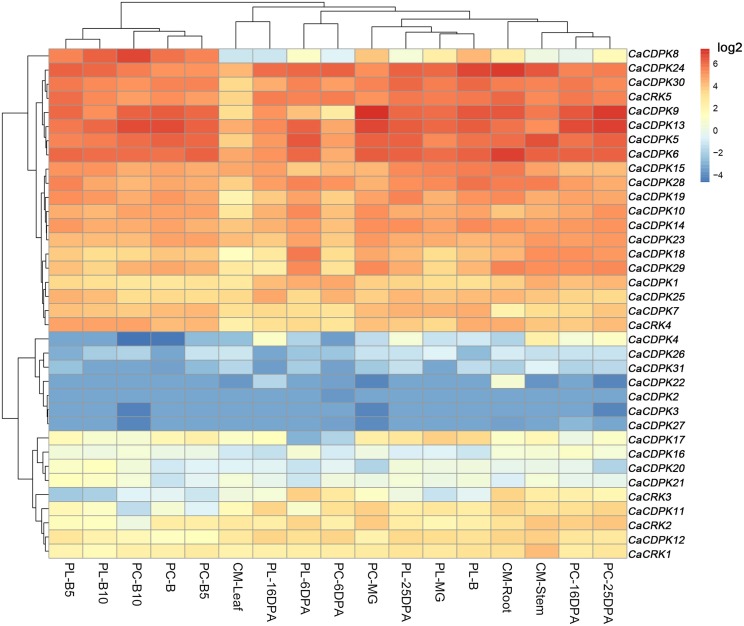
**Hierarchical clustering analyses of the expression of pepper CDPK and CRK genes in different pepper tissues**. The microarray data were obtained from pepper gene expression database under the series accession number (Kim et al., 2014b). PC, PL, and B indicate the pericarp, placenta, and breaker stages. DPA, days post-anthesis, CM, CM334 plants, MG, Mature Green. B, Break. B10, 10 days post-anthesis, respectively.

**Figure 5:**
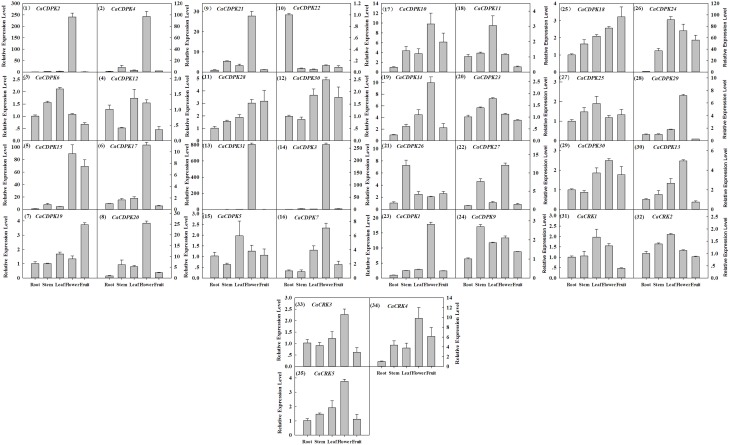
**Relative transcriptional expression levels of pepper CDPK and CRK genes in different tissues in pepper plants by quantitative real-time PCR**. The absolute transcript levels of the respective genes in the root were used as reference and set to a value of 1. The arrangement was according to the genes subfamilies.

Extensive studies have suggested a close relationship between gene expression and function. To investigate the possible roles of the CDPK genes in the pepper genome, the expression profiles of the 36 CDPK and CRK genes were analyzed in 17 tissues and developmental stages using microarray expression data recently published by Kim et al from CM334 pepper plants (Figure 4, Kim et al., 2014b). The results showed that most of the CDPK and CRK genes were expressed in different tissues and developmental stages in pepper. In addition, we analyzed the expression of all the pepper CDPK and CRK genes in different organs such as root, stem, leaf, flower, and fruit in plants of GZ03, an inbred line of pepper, under normal conditions using qRT-PCR (Figure 5). All of the CDPK genes except *CaCDPK16*, and all of CRK genes in the pepper genome, were expressed in at least one of the five tissues in non-stressed pepper plants, whereas *CaCDPK16* was not expressed in any of the five organs in non-stressed pepper plants. *CaCDPK22* was expressed constitutively in roots, but its expression levels in other organs were very low. *CaCDPK2, CaCDPK3, CaCDPK4*, and *CaCDPK31* were expressed constitutively only in flowers. All these data indicate that the members of the CDPK gene family and CRK genes might be involved in the growth and development of different tissues or organs of pepper plants.

### Expression profiles of pepper CDPK and CRK genes under high salt stress

**Figure 6:**
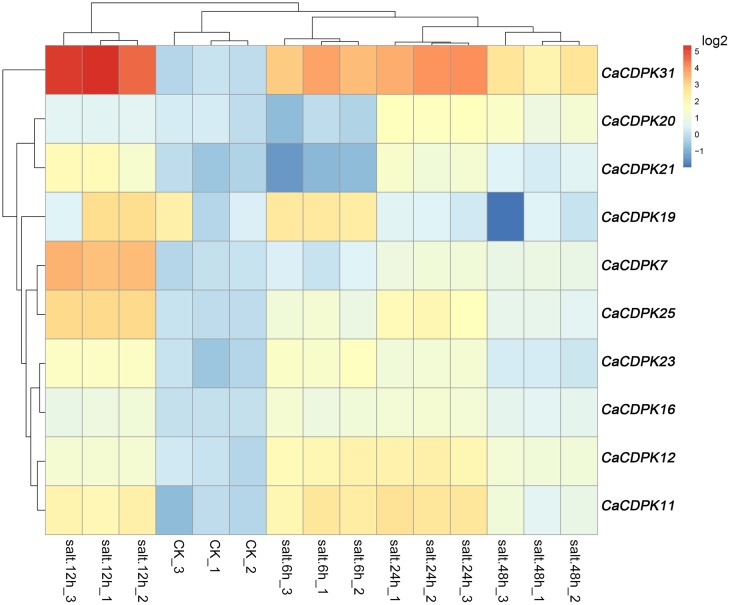
**Hierarchical clustering analyses on the relative expression of CDPK and CRK genes in the leaves of pepper plants challenged with salt stress**. The experiments were repeated three times, and the data were obtained by qPCR.

Salt stress is a major abiotic stress that exerts detrimental effects on crop growth and development, and causes heavy losses in crop yield. To test if CDPK genes in the pepper genome are involved in the response of pepper to salt stress, we examined the expression patterns of all the CDPKs and CRKs in pepper under salt stress treatment by qRT-PCR (Figure 6). Among the 31 CDPK and five CRK genes, 10 CDPK genes showed transcriptionally modified expression in response to salt stress, and all of the five CRK genes did not respond to salt stress. These 10 salt-responsive CDPK genes belonged to groups I (five genes), II (four genes), and III (one gene). It is noticeable that none of the salt stress-inducible CDPKs belong to group IV, suggesting that the response of pepper to salt stress might involve the reprogramming of multiple processes mediated by different CDPK genes. Similar expression profiles were found between *CaCDPK6, CaCDPK12*, and *CaCDPK23*, between *CaCDPK20* and *CaCDPK21*, between *CaCDPK11* and *CaCDPK19*, and between *CaCDPK7* and *CaCDPK25* under salt treatment, implying that multiple CDPK genes may act in a coordinated fashion to mediate the same process in pepper's response to salt stress.

### Expression profiles of pepper CDPK genes and CRK genes under high temperature stress

**Figure 7:**
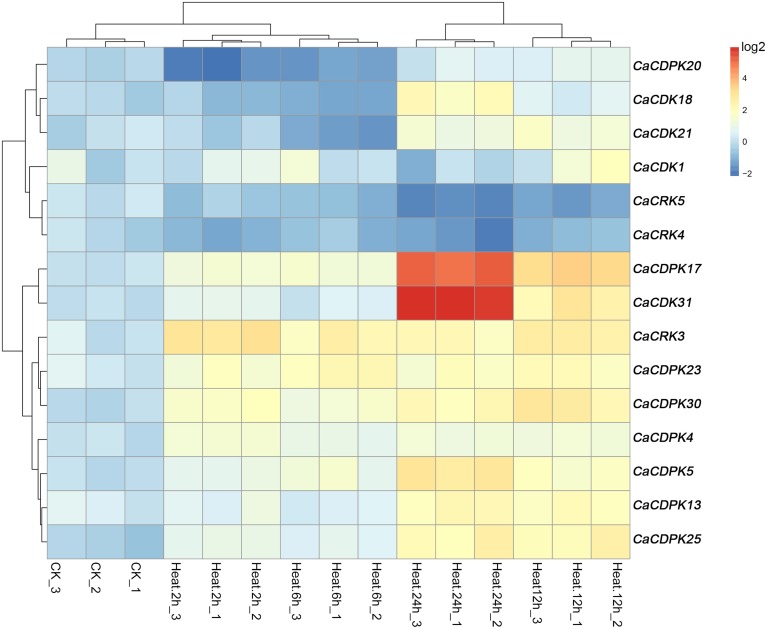
**Hierarchical clustering analyses on the relative expression of CDPK and CRK genes in leaves of pepper plants challenged with heat stress**. The experiments were repeated three times, and the data were obtained by qRT-PCR.

High temperature is another important abiotic stress that also exerts detrimental effects on crop growth and development and causes heavy losses in crop yield. To investigate if CDPK and CRK genes are involved in the response of pepper to heat stress, we examined their expression patterns by qRT-PCR under heat stress treatment. Out of the 35 CDPK and CRK genes, 13 CDPK and two CRK genes showed modified expression profiles in response to high temperature, suggesting that the response of pepper to high temperature may also include the reprogramming of multiple processes mediated by different CDPK and CRK genes. As showed in Figure 7, similar expression profiles among *CaCDPK25, CaCDPK13*, and *CaCDPK5, CaCDPK30, CaCDPK23* and *CaCRK3, CaCDPK18, CaCDPK20*, and *CaCDPK21*, and between *CaCDPK1* and *CaCDPK4, CaCRK4*, and *CaCRK5, CaCDPK31*, and *CaCDPK17* were found in response to high temperature treatment, implying that more than one CDPK genes may act in a coordinated fashion to mediate a single process in pepper's response to high temperature stress. The results also showed that most of the high temperature-responsive CDPK genes were upregulated in response to high temperature treatment; however, the transcripts of *CaCDPK18, CaCDPK20*, and *CaCDPK21* decreased at 2–6 hpt (hours post-treatment) and increased at 12–24 hpt, *CaCRK4*, and *CaCRK5* were downregulated in response to high temperature treatment, suggesting that these CDPK genes are involved in the response of pepper to high temperature, acting as positive or negative regulators.

### Expression profiles of pepper CDPK genes and CRK genes under *R. solanacearum* inoculation

**Figure 8:**
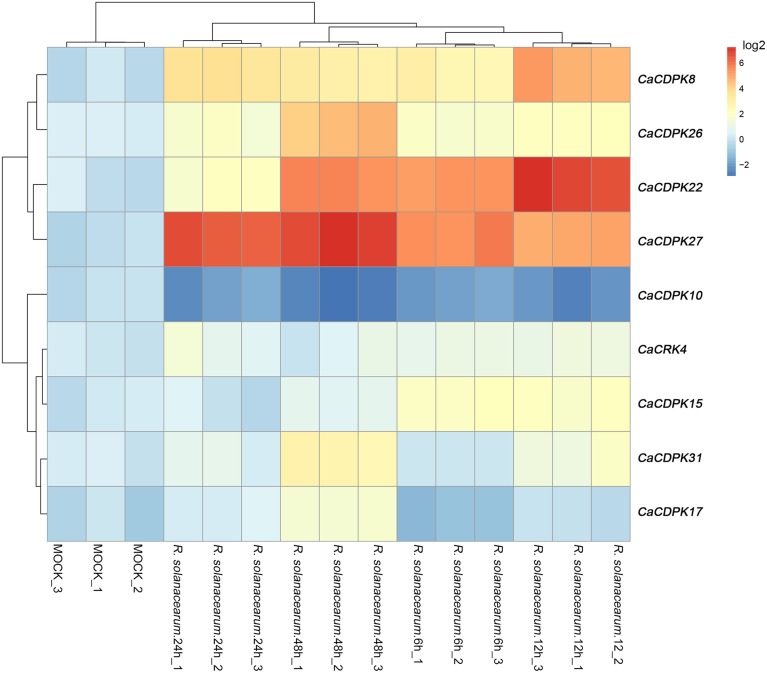
**Hierarchical clustering analyses on the relative expression of CDPK and CRK genes in the leaves of pepper plants challenged with ***Ralstonia solanacearum*** inoculation**. The experiments were repeated three times, and the data were obtained by qRT-PCR.

To test whether pepper CDPK and CRK genes are involved in the response to biotic stresses, the expression profiles of CDPK and CRK genes were monitored in pepper plants challenged with *R. solanacearum*, an important causal agent of pepper bacterial wilt worldwide. The results showed that, out of the 35 CDPK and five CRK genes in the pepper genome, 8 CDPK genes and one CRK gene showed altered expression patterns in response to *R. solanacearum* inoculation. As shown in Figure 8, eight CDPK genes were upregulated upon *R. solanacearum* inoculation, and only *CaCDPK10* was downregulated upon *R. solanacearum* inoculation. Among the eight upregulated CDPK genes, *CaCDPK27*, and *CaCDPK22* were dramatically induced by *R. solanacearum* inoculation. Similar expression profiles were found among *CaCDPK8, CaCDPK26*, and *CaCDPK31, CaCDPK15, CaCDPK17*, and *CaCRK4*, respectively, implying that these genes may act in a coordinated manner in mediating the response of pepper to *R. solanacearum* inoculation.

## Discussion

As typical member of *Solanaceae* and an agriculturally important vegetable, pepper is characterized by its large genome (~2.7 Gb), which is much larger than that of *Arabidopsis* (125 Mb) and rice (389 Mb). The 31 CDPK genes are much less than the number expected, compared to 34 CDPK genes in *Arabidopsis* (Cheng et al., 2002) and 31 in rice (Asano et al., 2005; Ray et al., 2007). Similar to the CDPK genes in other plant species such as *Arabidopsis*, rice, poplar, and cotton, the 31 CDPK genes were grouped into four subfamilies, with similar numbers of CDPK III and IV, and diverse numbers of CDPK I and II genes compared to those in other plant species, supporting the notion that the different numbers of CDPK genes in different plant species is contributed largely by the numbers of CDPK I or CDPK II genes that have evolved independently in different organisms (Liu et al., 2014). Like CDPK genes in other plants such as cotton and maize (Ferreira Neto et al., 2013; Zuo et al., 2013; Liu et al., 2014), the 31 CDPK genes and the five CRK genes identified in pepper genome are unevenly distribute on different chromosomes. Except for the typical CDPK domains, diversities in members of CDPK family among different plant species were observed. For example, less than half of the *Arabidopsis* CDPK genes had *Populus* homologs (Zuo et al., 2013). Similar sequences and exon-intron organization patterns were seen to be shared by members of the same subfamily of CDPK genes in the pepper genome and other plant species, suggesting the same origins of CDPK genes in different plant species. Similar to the duplicated CDPK gene pairs found in cotton and other plant species (Liu et al., 2014), eight duplicated CDPK gene pairs were found in the genome of pepper. Because more than 81% (~2.7 Gb) of the pepper genome was composed of different transposable elements (TEs), and the pepper genome is believed to have been expanded by a rapid amplification of retrotransposon elements (Kim et al., 2014b; Qin et al., 2014), the members of the CDPK family might have been expanded by amplification mediated by retrotransposon elements. Additionally, significant differences were observed in the deduced amino acid sequences of *CaCDPK6, CaCDPK7, CaCDPK10, CaCDPK12*, and *CaCDPK14* between CM334 and Chiltepin, and *CaCDPK31*, which is present in CM334, was not found in Chiltepin, suggesting active diversification of the members of CDPK family among different pepper germplasms. Further functional identification of these diversified CDPK genes might provide new insights into pepper immunity and other biological processes.

Accumulation evidences indicate that plant growth, development, and response to environmental stress are largely regulated at transcriptional level (Baena-González et al., 2007; Baena-González and Sheen, 2008; Rymen and Sugimoto, 2012; Buscaill and Rivas, 2014). In the present study, our data showed that CDPK genes in the pepper genome also exhibit specific spatial transcriptional expression patterns in different organs of pepper plants, and some CDPK genes in the pepper genome were found to be transcriptionally modified by cold, high temperature, and *R. solanacearum* inoculation, similarly to that in other plant species such as maize (Kong et al., 2013), cotton (Liu et al., 2014), and rice (Ray et al., 2007; Ye et al., 2009), suggesting the involvement of these CDPK genes in plant growth and responses to cold, heat stress, and pathogens. Interestingly, similar responses to salt stress were found in pepper CDPK genes and their orthologues in *Arabidopsis*. For example, salt stress-responsive *CaCDPK31* shared 60% sequence identity to *AtCPK11*, a positive regulator of salt stress tolerance (Zhu et al., 2007). *CaCDPK19* and *CaCDPK12* shared 56% sequence identities with *AtCPK4*, which has previously been shown to be involved in salt stress (Zhu et al., 2007). *CaCDPK23* shared 70.89% sequence identity to *AtCPK6*, whose overexpression enhanced the tolerance of transgenic plants to salt/drought stresses, whereas *atcpk6* mutant plants display no obvious phenotypes (Xu et al., 2010). *CaCDPK7* shared 58.22% sequence identity to *AtCPK21/23*, and *atcpk21*, and *atcpk23* mutants showed increased tolerance to hyperosmotic stress, drought, and salt stresses (Ma and Wu, 2007; Franz et al., 2011). These results suggest a close relationship between the sequence of CDPK genes and their function in the salt stress response. Transcriptional reprogrammings were previously assayed comparatively against different biotic and abiotic stresses using stress resistant or tolerant and stress sensitive plant accessions, differences observed between the resistant or tolerant and sensitive plant accessions against the same stress are usually temporal and quantitative not qualitative (Maleck et al., 2000; Tao et al., 2003; Puckette et al., 2008; Tsuda et al., 2009; Thomma et al., 2011; Pons Puig et al., 2015), especially between compatible and incompatible plant-pathogen interactions or ETI (effector triggered immunity) and PTI (PAMP triggered immunity) (Maleck et al., 2000; Tao et al., 2003; Thomma et al., 2011), although the defense reactions are usually faster and stronger in ETI or incompatible plant-pathogen interactions than in PTI or compatible plant-pathogen interactions. The overexpression of CDPK genes such as OsCPK9 (Wei et al., 2014), OsCPK12 (Asano et al., 2012), and ZmCPK4 (Jiang et al., 2013) transcripionally upregulated generally leads to enhanced stress tolerance/resistance in transgenic plants, suggesting potential values of the CDPK genes upregulated by stresses in the present study in genetic improvement of crop stress resistance/tolerance.

There were several novel findings in the present study that are worth pointing out. First, upon exposure to cold, heat stress and *R*. *solanacearum* inoculation, the expression of 10, 12, and 8 CDPK genes were transcriptionally modified by salt, heat, and *R. solanacearum* inoculation, respectively. Similar observations were previously published in rice, in which more than one CDPKs such as *OsCPK4, OsCPK12, and OsCPK21* were seen to function as positive regulators of the salt response (Asano et al., 2011, 2012; Campo et al., 2014), and *OsCDPK7, OsCPK10*, and *OsCPK12* act as positive regulators of disease resistance (Mall et al., 2011; Fu et al., 2013). Among the CDPK genes responsive to heat and *R. solanacearum* inoculation, three CDPK genes (CaCDPK18, -20, and -21) were downregulated by heat stress, and CaCDPK10 was downregulated by *R. solanacearum* inoculation, indicating that the response of pepper to heat and *R. solanacearum* inoculation are fine tuned by various CDPK genes, with the majority of them acting as positive regulators and a small number of them acting as negative regulators. Similar observations that numerous CDPK genes were transcriptionally modified by one stress were found in the responses of cotton CDPK genes to cold (Liu et al., 2014), of poplar CDPK genes to drought (Zuo et al., 2013), and of rice CDPK genes to cold and salt (Ray et al., 2007). Some CDPK genes in the pepper genome shared similar expression profiles in response to a specific stress, for example, similar expression levels were observed between *CaCDPK25, CaCDPK13, and CaCDPK5; CaCDPK30 and CaCDPK23; CaCDPK18, CaCDPK20, and CaCDPK21; CaCDPK1 and CaCDPK4; and CaCDPK31 and CaCDPK17* upon the exposure to heat stress. In contrast, *CaCDPK8, CaCDPK26*, and *CaCDPK31; CaCDPK15* and *CaCDPK17;* and *CaCDPK27* and *CaCDPK22* showed similar expression in pepper plants challenged with *R. solanacearum* inoculation, implying coordinated regulation of a process in plant response to a specific stress by these CDPK genes. Secondly, although the majority of CDPK genes were specifically expressed under treatment with salt, heat, and *R. solanacearum* inoculation, some CDPK genes were found to respond to more than one stress. For example, *CaCDPK23, CaCDPK25*, and *CaCDPK31* were upregulated by both salt and heat stress, and *CaCDPK17* and *CaCDPK31* were upregulated by both heat and *R. solanacearum* inoculation, and *CaCDPK31* was upregulated by salt, heat, and *R. solanacearum* inoculation. In contrast, *CaCDPK20* and *CaCDPK21* were upregulated by salt, but downregulated by heat stress. These results suggest that crosstalk between pepper responses to salt and heat, to salt and *R. solanacearum* inoculation, as well as to heat and *R. solanacearum* inoculation is mediated by CDPK genes. In particular, *CaCDPK31* acts in convergence in the response of pepper to salt, heat, and *R. solanacearum* inoculation. Similar crosstalk between plant responses to biotic and abiotic stresses have been found to be mediated by CDPK genes; for example, *PaCDPK1* was induced by cold, wounding, and pathogen challenge (Tsai et al., 2007), *TaCPK7* was induced by drought, salt, cold, and hydrogen peroxide (Geng et al., 2011), and *OsCPK12* in rice functions in multiple signaling pathways, positively regulating salt tolerance and negatively modulating blast resistance (Asano et al., 2012).

Collectively, the present study identified 31 CDPK genes and five closely related kinase genes in the pepper genome, which can be phylogenetically classified into four distinct subfamilies and are unevenly distributed across nine chromosomes. Conserved sequence and exon-intron structures are shared by CDPKs of pepper within the same subfamily, and the expansion of CaCDPK family in pepper was due to segmental duplication events. Five CDPK genes in *CM334* were found to be mutated in Chiltepin, and one CDPK present in *CM334* was lost in *Chiltepin*. The majority of the CDPK and CRK genes showed differential expression in different pepper tissues and organs. In addition, 10, 12, and 8 CDPK genes were transcriptionally modified by salt, heat, and *R. solanacearum* inoculation, respectively; the majority of these genes responded specifically to one stress, whereas a small number of these genes responded synergistically to two or three stresses, suggesting that these CDPK genes might be involved in the specific or synergistic response of pepper to salt, heat, and *R. solanacearum* inoculation. Our results will prove helpful for the functional characterization of pepper CDPK and its closely related gene families.

### Conflict of interest statement

The authors declare that the research was conducted in the absence of any commercial or financial relationships that could be construed as a potential conflict of interest.

## References

[B1] Asano T. Hakata M. Nakamura H. Aoki N. Komatsu S. Ichikawa H.. (2011). Functional characterisation of OsCPK21, a calcium-dependent protein kinase that confers salt tolerance in rice. Plant Mol. Biol. 75, 179–191. 10.1007/s11103-010-9717-1 21136139

[B2] Asano T. Hayashi N. Kobayashi M. Aoki N. Miyao A. Mitsuhara I.. (2012). A rice calcium-dependent protein kinase OsCPK12 oppositely modulates salt-stress tolerance and blast disease resistance. Plant J. 69, 26–36. 10.1111/j.1365-313X.2011.04766.x 21883553

[B3] Asano T. Tanaka N. Yang G. Hayashi N. Komatsu S. (2005). Genome-wide identification of the rice calcium-dependent protein kinase and its closely related kinase gene families: comprehensive analysis of the CDPKs gene family in rice. Plant Cell Physiol. 46, 356–366. 10.1093/pcp/pci035 15695435

[B4] Baena-Gonzalez E. Sheen J. (2008). Convergent energy and stress signaling. Trends Plant Sci. 13, 474–482. 10.1016/j.tplants.2008.06.006 18701338 PMC3075853

[B5] Baena-González E. Rolland F. Thevelein J. M. Sheen J. (2007). A central integrator of transcription networks in plant stress and energy signalling. Nature 448, 938–942. 10.1038/nature06069 17671505

[B6] Buscaill P. Rivas S. (2014). Transcriptional control of plant defence responses. Curr. Opin. Plant Biol. 20, 35–46. 10.1016/j.pbi.2014.04.004 24840291

[B7] Campo S. Baldrich P. Messeguer J. Lalanne E. Coca M. San Segundo B. (2014). Overexpression of a calcium-dependent protein kinase confers salt and drought tolerance in rice by preventing membrane lipid peroxidation. Plant Physiol. 165, 688–704. 10.1104/pp.113.230268 24784760 PMC4044838

[B8] Chang W. J. Su H. S. Li W. J. Zhang Z. L. (2009). Expression profiling of a novel calcium-dependent protein kinase gene, LeCPK2, from tomato (Solanum lycopersicum) under heat and pathogen-related hormones. Biosci. Biotechnol. Biochem. 73, 2427–2431. 10.1271/bbb.90385 19897910

[B9] Chen F. Fasoli M. Tornielli G. B. Dal Santo S. Pezzotti M. Zhang L.. (2013). The evolutionary history and diverse physiological roles of the grapevine calcium-dependent protein kinase gene family. PLoS ONE 8:e80818. 10.1371/journal.pone.0080818 24324631 PMC3855637

[B10] Cheng S. H. Willmann M. R. Chen H. C. Sheen J. (2002). Calcium signaling through protein kinases. The Arabidopsis calcium-dependent protein kinase gene family. Plant Physiol. 129, 469–485. 10.1104/pp.005645 12068094 PMC1540234

[B11] Chico J. M. Raices M. Téllez-Iñón M. T. Ulloa R. M. (2002). A calcium-dependent protein kinase is systemically induced upon wounding in tomato plants. Plant Physiol. 128, 256–270. 10.1104/pp.010649 11788771 PMC148989

[B12] Chmielowska-Bak J. Lefévre I. Lutts S. Deckert J. (2013). Short term signaling responses in roots of young soybean seedlings exposed to cadmium stress. J. Plant Physiol. 170, 1585–1594. 10.1016/j.jplph.2013.06.019 23942356

[B13] Dammann C. Ichida A. Hong B. Romanowsky S. M. Hrabak E. M. Harmon A. C.. (2003). Subcellular targeting of nine calcium-dependent protein kinase isoforms from Arabidopsis. Plant Physiol. 132, 1840–1848. 10.1104/pp.103.020008 12913141 PMC181270

[B14] Dang F. F. Wang Y. N. Yu L. Eulgem T. Lai Y. Liu Z. Q.. (2013). CaWRKY40, a WRKY protein of pepper, plays an important role in the regulation of tolerance to heat stress and resistance to *Ralstonia solanacearum* infection. Plant Cell Environ. 36, 757–774. 10.1111/pce.12011 22994555

[B15] Ding Y. Cao J. Ni L. Zhu Y. Zhang A. Tan M.. (2013). ZmCPK11 is involved in abscisic acid-induced antioxidant defence and functions upstream of ZmMPK5 in abscisic acid signalling in maize. J. Exp. Bot. 64, 871–884. 10.1093/jxb/ers366 23268839 PMC3580805

[B16] Estruch J. J. Kadwell S. Merlin E. Crossland L. (1994). Cloning and characterization of a maize pollen-specific calcium-dependent calmodulin-independent protein kinase. Proc. Natl. Acad. Sci. U.S.A. 91, 8837–8841. 10.1073/pnas.91.19.8837 8090732 PMC44701

[B17] Ferreira Neto J. R. Pandolfi V. Guimaraes F. C. Benko-Iseppon A. M. Romero C. Silva R. L.. (2013). Early transcriptional response of soybean contrasting accessions to root dehydration. PLoS ONE 8:e83466. 10.1371/journal.pone.0083466 24349513 PMC3861472

[B18] Franz S. Ehlert B. Liese A. Kurth J. Cazalé A. C. Romeis T. (2011). Calcium-dependent protein kinase CPK21 functions in abiotic stress response in *Arabidopsis thaliana*. Mol. Plant 4, 83–96. 10.1093/mp/ssq064 20978086

[B19] Frattini M. Morello L. Breviario D. (1999). Rice calcium-dependent protein kinase isoforms OsCDPK2 and OsCDPK11 show different responses to light and different expression patterns during seed development. Plant Mol. Biol. 41, 753–764. 10.1023/A:1006316422400 10737140

[B20] Freymark G. Diehl T. Miklis M. Romeis T. Panstruga R. (2007). Antagonistic control of powdery mildew host cell entry by barley calcium-dependent protein kinases (CDPKs). Mol. Plant Microbe Interact. 20, 1213–1221. 10.1094/MPMI-20-10-1213 17918623

[B21] Fu L. Yu X. An C. (2013). Overexpression of constitutively active OsCPK10 increases Arabidopsis resistance against *Pseudomonas syringae* pv. tomato and rice resistance against Magnaporthe grisea. Plant Physiol. Biochem. 73, 202–210. 10.1016/j.plaphy.2013.10.004 24141028

[B22] Geng S. Zhao Y. Tang L. Zhang R. Sun M. Guo H.. (2011). Molecular evolution of two duplicated CDPK genes CPK7 and CPK12 in grass species: a case study in wheat (*Triticum aestivum* L.). Gene 475, 94–103. 10.1016/j.gene.2010.12.015 21241786

[B23] Harmon A. C. (2003). Calcium-regulated protein kinases of plants. Gravit. Space Biol. Bull. 16, 83–90. 12959135

[B24] Harper J. F. Sussman M. R. Schaller G. E. Putnam-Evans C. Charbonneau H. Harmon A. C. (1991). A calcium-dependent protein kinase with a regulatory domain similar to calmodulin. Science 252, 951–954. 10.1126/science.1852075 1852075

[B25] Hrabak E. M. Chan C. W. Gribskov M. Harper J. F. Choi J. H. Halford N.. (2003). The Arabidopsis CDPK-SnRK superfamily of protein kinases. Plant Physiol. 132, 666–680. 10.1104/pp.102.011999 12805596 PMC167006

[B26] Huang T. L. Nguyen Q. T. Fu S. F. Lin C. Y. Chen Y. C. Huang H. J. (2012). Transcriptomic changes and signalling pathways induced by arsenic stress in rice roots. Plant Mol. Biol. 80, 587–608. 10.1007/s11103-012-9969-z 22987115

[B27] Ivashuta S. Liu J. Liu J. Lohar D. P. Haridas S. Bucciarelli B.. (2005). RNA interference identifies a calcium-dependent protein kinase involved in Medicago truncatula root development. Plant Cell 17, 2911–2921. 10.1105/tpc.105.035394 16199614 PMC1276019

[B28] Jain M. Pathak B. P. Harmon A. C. Tillman B. L. Gallo M. (2011). Calcium dependent protein kinase (CDPK) expression during fruit development in cultivated peanut (Arachis hypogaea) under Ca(2)(+)-sufficient and -deficient growth regimens. J. Plant Physiol. 168, 2272–2277. 10.1016/j.jplph.2011.07.005 21862174

[B29] Jaworski K. Pawelek A. Kopcewicz J. Szmidt-Jaworska A. (2012). The calcium-dependent protein kinase (PnCDPK1) is involved in Pharbitis nil flowering. J. Plant Physiol. 169, 1578–1585. 10.1016/j.jplph.2012.05.025 22840323

[B30] Jiang S. Zhang D. Wang L. Pan J. Liu Y. Kong X.. (2013). A maize calcium-dependent protein kinase gene, ZmCPK4, positively regulated abscisic acid signaling and enhanced drought stress tolerance in transgenic Arabidopsis. Plant Physiol. Biochem. 71, 112–120. 10.1016/j.plaphy.2013.07.004 23911729

[B31] Kim M. K. Seo J. K. Kwak H. R. Kim J. S. Kim K. H. Cha B. J.. (2014a). Molecular genetic analysis of cucumber mosaic virus populations infecting pepper suggests unique patterns of evolution in Korea. Phytopathology 104, 993–1000. 10.1094/PHYTO-10-13-0275-R 25116642

[B32] Kim S. Park M. Yeom S. I. Kim Y. M. Lee J. M. Lee H. A.. (2014b). Genome sequence of the hot pepper provides insights into the evolution of pungency in Capsicum species. Nat. Genet. 46, 270–278. 10.1038/ng.2877 24441736

[B33] Kobayashi M. Ohura I. Kawakita K. Yokota N. Fujiwara M. Shimamoto K.. (2007). Calcium-dependent protein kinases regulate the production of reactive oxygen species by potato NADPH oxidase. Plant Cell 19, 1065–1080. 10.1105/tpc.106.048884 17400895 PMC1867354

[B34] Kong X. Lv W. Jiang S. Zhang D. Cai G. Pan J.. (2013). Genome-wide identification and expression analysis of calcium-dependent protein kinase in maize. BMC Genomics 14:433. 10.1186/1471-2164-14-433 23815483 PMC3704972

[B35] Lee S. S. Cho H. S. Yoon G. M. Ahn J. W. Kim H. H. Pai H. S. (2003). Interaction of NtCDPK1 calcium-dependent protein kinase with NtRpn3 regulatory subunit of the 26S proteasome in *Nicotiana tabacum*. Plant J. 33, 825–840. 10.1046/j.1365-313X.2003.01672.x 12609025

[B36] Li A. L. Zhu Y. F. Tan X. M. Wang X. Wei B. Guo H. Z.. (2008). Evolutionary and functional study of the CDPK gene family in wheat (*Triticum aestivum* L.). Plant Mol. Biol. 66, 429–443. 10.1007/s11103-007-9281-5 18185910

[B37] Li R. J. Hua W. Lu Y. T. (2006). Arabidopsis cytosolic glutamine synthetase AtGLN1;1 is a potential substrate of AtCRK3 involved in leaf senescence. Biochem. Biophys. Res. Commun. 342, 119–126. 10.1016/j.bbrc.2006.01.100 16472779

[B38] Liu W. Li W. He Q. Daud M. K. Chen J. Zhu S. (2014). Genome-wide survey and expression analysis of calcium-dependent protein kinase in *Gossypium raimondii*. PLoS ONE 9:e98189. 10.1371/journal.pone.0098189 24887436 PMC4041719

[B39] Livak K. J. Schmittgen T. D. (2001). Analysis of relative gene expression data using real-time quantitative PCR and the 2(-Delta Delta C(T)) Method. Methods 25, 402–408. 10.1006/meth.2001.1262 11846609

[B40] Ludwig A. A. Romeis T. Jones J. D. (2004). CDPK-mediated signalling pathways: specificity and cross-talk. J. Exp. Bot. 55, 181–188. 10.1093/jxb/erh008 14623901

[B41] Ma S. Y. Wu W. H. (2007). AtCPK23 functions in Arabidopsis responses to drought and salt stresses. Plant Mol. Biol. 65, 511–518. 10.1007/s11103-007-9187-2 17541706

[B42] Maleck K. Levine A. Eulgem T. Morgan A. Schmid J. Lawton K. A.. (2000). The transcriptome of *Arabidopsis thaliana* during systemic acquired resistance. Nat. Genet. 26, 403–410. 10.1038/82521 11101835

[B43] Mall T. K. Dweikat I. Sato S. J. Neresian N. Xu K. Ge Z.. (2011). Expression of the rice CDPK-7 in sorghum: molecular and phenotypic analyses. Plant Mol. Biol. 75, 467–479. 10.1007/s11103-011-9741-9 21318369

[B44] Martín M. L. Busconi L. (2000). Membrane localization of a rice calcium-dependent protein kinase (CDPK) is mediated by myristoylation and palmitoylation. Plant J. 24, 429–435. 10.1046/j.1365-313x.2000.00889.x 11115124

[B45] Martín M. L. Busconi L. (2001). A rice membrane-bound calcium-dependent protein kinase is activated in response to low temperature. Plant Physiol. 125, 1442–1449. 10.1104/pp.125.3.1442 11244123 PMC65622

[B46] Pons Puig C. Dagar A. Marti Ibanez C. Singh V. Crisosto C. H. Friedman H.. (2015). Pre-symptomatic transcriptome changes during cold storage of chilling sensitive and resistant peach cultivars to elucidate chilling injury mechanisms. BMC Genomics 16:245. 10.1186/s12864-015-1395-6 25887353 PMC4391166

[B47] Puckette M. C. Tang Y. Mahalingam R. (2008). Transcriptomic changes induced by acute ozone in resistant and sensitive Medicago truncatula accessions. BMC Plant Biol. 8:46. 10.1186/1471-2229-8-46 18433496 PMC2395263

[B48] Qin C. Yu C. Shen Y. Fang X. Chen L. Min J.. (2014). Whole-genome sequencing of cultivated and wild peppers provides insights into Capsicum domestication and specialization. Proc. Natl. Acad. Sci. U.S.A. 111, 5135–5140. 10.1073/pnas.1400975111 24591624 PMC3986200

[B49] Ray S. Agarwal P. Arora R. Kapoor S. Tyagi A. K. (2007). Expression analysis of calcium-dependent protein kinase gene family during reproductive development and abiotic stress conditions in rice (*Oryza sativa* L. ssp. indica). Mol. Genet. Genomics 278, 493–505. 10.1007/s00438-007-0267-4 17636330

[B50] Romeis T. Herde M. (2014). From local to global: CDPKs in systemic defense signaling upon microbial and herbivore attack. Curr. Opin. Plant Biol. 20C, 1–10. 10.1016/j.pbi.2014.03.002 24681995

[B51] Rymen B. Sugimoto K. (2012). Tuning growth to the environmental demands. Curr. Opin. Plant Biol. 15, 683–690. 10.1016/j.pbi.2012.07.005 22902170

[B52] Saijo Y. Hata S. Kyozuka J. Shimamoto K. Izui K. (2000). Over-expression of a single Ca2+-dependent protein kinase confers both cold and salt/drought tolerance on rice plants. Plant J. 23, 319–327. 10.1046/j.1365-313x.2000.00787.x 10929125

[B53] Shen Y. Y. Duan C. Q. Liang X. E. Zhang D. P. (2004). Membrane-associated protein kinase activities in the developing mesocarp of grape berry. J. Plant Physiol. 161, 15–23. 10.1078/0176-1617-01038 15002660

[B54] Tamura K. Peterson D. Peterson N. Stecher G. Nei M. Kumar S. (2011). MEGA5: molecular evolutionary genetics analysis using maximum likelihood, evolutionary distance, and maximum parsimony methods. Mol. Biol. Evol. 28, 2731–2739. 10.1093/molbev/msr121 21546353 PMC3203626

[B55] Tao Y. Xie Z. Chen W. Glazebrook J. Chang H. S. Han B.. (2003). Quantitative nature of Arabidopsis responses during compatible and incompatible interactions with the bacterial pathogen *Pseudomonas syringae*. Plant Cell 15, 317–330. 10.1105/tpc.007591 12566575 PMC141204

[B56] Thomma B. P. Nürnberger T. Joosten M. H. (2011). Of PAMPs and effectors: the blurred PTI-ETI dichotomy. Plant Cell 23, 4–15. 10.1105/tpc.110.082602 21278123 PMC3051239

[B57] Tsai T. M. Chen Y. R. Kao T. W. Tsay W. S. Wu C. P. Huang D. D.. (2007). PaCDPK1, a gene encoding calcium-dependent protein kinase from orchid, Phalaenopsis amabilis, is induced by cold, wounding, and pathogen challenge. Plant Cell Rep. 26, 1899–1908. 10.1007/s00299-007-0389-5 17593367

[B58] Tsuda K. Sato M. Stoddard T. Glazebrook J. Katagiri F. (2009). Network properties of robust immunity in plants. PLoS Genet. 5:e1000772. 10.1371/journal.pgen.1000772 20011122 PMC2782137

[B59] Vivek P. J. Tuteja N. Soniya E. V. (2013). CDPK1 from ginger promotes salinity and drought stress tolerance without yield penalty by improving growth and photosynthesis in *Nicotiana tabacum*. PLoS ONE 8:e76392. 10.1371/journal.pone.0076392 24194837 PMC3806807

[B60] Wan B. Lin Y. Mou T. (2007). Expression of rice Ca(2+)-dependent protein kinases (CDPKs) genes under different environmental stresses. FEBS Lett. 581, 1179–1189. 10.1016/j.febslet.2007.02.030 17336300

[B61] Wei S. Hu W. Deng X. Zhang Y. Liu X. Zhao X.. (2014). A rice calcium-dependent protein kinase OsCPK9 positively regulates drought stress tolerance and spikelet fertility. BMC Plant Biol. 14:133. 10.1186/1471-2229-14-133 24884869 PMC4036088

[B62] Wurzinger B. Mair A. Pfister B. Teige M. (2011). Cross-talk of calcium-dependent protein kinase and MAP kinase signaling. Plant Signal. Behav. 6, 8–12. 10.4161/psb.6.1.14012 21248475 PMC3121996

[B63] Xu J. Tian Y. S. Peng R. H. Xiong A. S. Zhu B. Jin X. F.. (2010). AtCPK6, a functionally redundant and positive regulator involved in salt/drought stress tolerance in Arabidopsis. Planta 231, 1251–1260. 10.1007/s00425-010-1122-0 20217124

[B64] Ye S. Wang L. Xie W. Wan B. Li X. Lin Y. (2009). Expression profile of calcium-dependent protein kinase (CDPKs) genes during the whole lifespan and under phytohormone treatment conditions in rice (*Oryza sativa* L. ssp. indica). Plant Mol. Biol. 70, 311–325. 10.1007/s11103-009-9475-0 19263224

[B65] Yoon G. M. Cho H. S. Ha H. J. Liu J. R. Lee H. S. (1999). Characterization of NtCDPK1, a calcium-dependent protein kinase gene in *Nicotiana tabacum*, and the activity of its encoded protein. Plant Mol. Biol. 39, 991–1001. 10.1023/A:1006170512542 10344204

[B66] Zhou R. Q. Nie K. Huang H. C. Hu S. J. Zhou Z. Y. Luo H. L. (2009). Phylogenetic analysis of Mycoplasma suis isolates based on 16S rRNA gene sequence in China. Vet. Res. Commun. 33, 855–863. 10.1007/s11259-009-9234-3 19590972

[B67] Zhu S. Y. Yu X. C. Wang X. J. Zhao R. Li Y. Fan R. C.. (2007). Two calcium-dependent protein kinases, CPK4 and CPK11, regulate abscisic acid signal transduction in Arabidopsis. Plant Cell 19, 3019–3036. 10.1105/tpc.107.050666 17921317 PMC2174700

[B68] Zuo R. Hu R. Chai G. Xu M. Qi G. Kong Y.. (2013). Genome-wide identification, classification, and expression analysis of CDPK and its closely related gene families in poplar (*Populus trichocarpa*). Mol. Biol. Rep. 40, 2645–2662. 10.1007/s11033-012-2351-z 23242656

