# Supplementary material for: Genome-wide identification and expression analysis of calcium-dependent protein kinase and its closely related kinase genes in Capsicum annuum
Source: Front Plant Sci. 2015 Sep 15;6:737. doi: 10.3389/fpls.2015.00737 (PMC4584942; doi:10.3389/fpls.2015.00737)
Supplement: Supplementary file 1 [file Table1.DOC]

Table S1. The qRT-PCR primers of CDPK and CRK genes

| Name | Name | Name | Left primer (5ʹ to 3ʹ) | Right primer (5ʹ to 3ʹ) |
| --- | --- | --- | --- | --- |
| CaCDPK1 | Capang01g001337 | CA01g10370 | GTGTGAAGCCGGGTAAATC | GTGTGAAGCCGGGTAAATC |
| CaCDPK2 | Capang01g001406 | CA01g08810 | ATAGGACCTAAGTTAGGCAAC | CTAGTAGGATCACTGCTACC |
| CaCDPK3 | Capang01g001476 | CA01g10500 | CTCTAGAGGAGAAGAACAAC | GCTAGGGGATGTATGAGGAG |
| CaCDPK4 | Capang01g001806 | CA10g17030 | AGGGGTATTAAAGAACCTCC | CTTTTCCCTCTCTATCTTCACC |
| CaCDPK5 | Capang01g005134 | CA07g17490 | AATGGGCACCGTGGATCAAG | CTTGGTGGTGGCCTCACAGGC |
| CaCDPK6 | Capang01g005424 | CA00g76700 | GCCGTGGATCTTTCCGAGCC | GGTCTTATGACCCAGAACGC |
| CaCDPK7 | Capang02g000275 | CA02g03450 | GCACAACGAGATGGTAAAGGG | TTGCGGTTGTGGCAGTGGC |
| CaCDPK8 | Capang02g002320 | CA00g55450 | TCTGCTAGCTCAAAAGTTAG | GCTTATTGCAATGAGACCCCTC |
| CaCDPK9 | Capang03g001022 | CA00g37630 | CGCCTGCATAAGACCAGAAG | TAAGTTACTCCGAATTCACC |
| CaCDPK10 | Capang03g002819 | CA03g10950 | GAGCAAAACTAAACCAGCAG | ACTTGCTTGAGGCCTCGTGG |
| CaCDPK11 | Capang04g000135 | CA04g16340 | AGTAAAGCATGTTGTTGTCC | CGACGATATTGGTGCTGGTG |
| CaCDPK12 | Capang04g001808 | CA04g08430 | CGCAGAGAATAGCACAACAC | TTGGGGATTGATTTGCAGGC |
| CaCDPK13 | Capang04g002071 | CA04g03980 | CAAGTTCCAAAGTTAGTGGCTC | GCTGCTTTTGAGGTTTCCCTGGG |
| CaCDPK14 | Capang04g002230 | CA04g01680 | GCAAGAAATATACCCAACAAG | TGCCTGTTGCTGTGGCTTCG |
| CaCDPK15 | Capang05g000414 | CA05g03200 | GAGCCTTCGCCTTTCAAGATTTTT | CTGATGTGCATTGAAACAGCAAGGT |
| CaCDPK16 | Capang06g000671 | CA06g16860 | GATCAAGAAAGTTGTTCCAAG | CCTCTTCCTAACTCCTTCCC |
| CaCDPK17 | Capang06g001318 | CA06g11830 | GCCAAAAAGAGACCTCCAATATC | GATATGTTGTACCAAATTGCCC |
| CaCDPK18 | Capang06g002308 | CA10g13810 | GATCTCCGGCAGCTGTAGC | GTCACGCCAAATTCGCCTC |
| CaCDPK19 | Capang07g000013 | CA00g67180 | CCAAAAACATCATCTTTATC | CTCCCTCCAAACATCCTC |
| CaCDPK20 | Capang07g000770 | CA10g17200 | GGAGGTGAAACAGAATAAGCC | CCCTTGTCCTAACTTCCTCCC |
| CaCDPK21 | Capang07g000773 | CA10g17230 | GTGTAGGACCCAAATTGGC | AGATCCATCCGTTTTCGATG |
| CaCDPK22 | Capang08g000525 | CA00g32950 | GTTCATGAAAAGATCTCGAAG | ATGATAACCTGCTCAGCCCC |
| CaCDPK23 | Capang08g001300 | CA00g24640 | TAGCACCTTATCTTCCGACC | ACGCCGAATTGACCGCGGCC |
| CaCDPK24 | Capang10g002105 | CA00g13850 | GCAGTACCAAAAACTTCAGA | TACGTAACCCCAAATTCACC |
| CaCDPK25 | Capang11g000778 | CA11g11090 | CCAAACCCTTTTGCTCTTG | CCTAGCTCATGACCAAGATC |
| CaCDPK26 | Capang12g000076 | CA12g21580 | CACACCATCAAAAAGTCCTCC | TTCCTAGGGTGTATGTTGCC |
| CaCDPK27 | Capang12g000830 | CA11g11120 | GTTGTTCAAGGGGCCAACC | GATTTTGGTGGTGTAACTGGG |
| CaCDPK28 | Capang12g001828 | CA12g07230 | CGCCGGATGATACTGCTTCCAC | CATTGTTATATGTTCTGGTGGC |
| CaCDPK29 | Capang00g003107 | CA09g10260 | GCAGATCTCCGGCAGCTGTTG | ATCTACCAAATACCTCTCC |
| CaCDPK30 | Capang00g003951 | CA10g09970 | GCCGTGGATCTATTGGAGGC | GCCTGGTTACTGCTTGTACG |
| CaCDPK31 |  | CA00g35300 | CGGGACCCACGTTGAACAAAG | GTGCCATCATTGCCATCGCC |
| CaCRK | CaCRK |  |  |  |
| CaCRK1 | Capang01g000496 | CA08g12420 | CCTCAAAGCCTCCAAGACCC | GAAGCAGAAGAGTGACGGGC |
| CaCRK2 | Capang02g000628 | CA02g02040 | TCAAAACCTCCCCCTGAACC | GGGAGTAGAATTTGCCGGAG |
| CaCRK3 | Capang02g002836 | CA02g23060 | ACCCTCAAATTACTCCGGCG | ACTTGGACTGTAGAAAGGG |
| CaCRK4 | Capang03g001828 | CA03g19510 | CCACCGCCCTAAACCACCGC | AGACGGTGGTGGAAATGGCC |
| CaCRK5 | Capang03g002325 | CA03g15750 | GCAACAGCAACACTGAAACA | CTGATTATCCCAAACTGCCC |
