# Supplementary material for: Genome-wide identification and expression analysis of calcium-dependent protein kinase and its closely related kinase genes in Capsicum annuum
Source: Front Plant Sci. 2015 Sep 15;6:737. doi: 10.3389/fpls.2015.00737 (PMC4584942; doi:10.3389/fpls.2015.00737)
Supplement: Supplementary file 2 [file DataSheet1.DOC]

>CaCDPK1

MGNCCVKPGKSAEKKNKKVEKPNPFAIDYGGAKHVNKLVVLKDPTGQNIRDKYDLGLELGRGEFGVTYLCTEVDTDEKYACKSISKKKLRTAVDIDDVRREVEIMKHLPKHPNVVTLKDTYEDDNAVHIVMELCEGGELFDRIVARGHYTERAAAGILKTVVEVVLMCHRHGVMHRDLKPENFLFGNKKETAPLKAIDFGLSVFFKPGERFNEIVGSPYYMAPEVLKRNYGPEVDIWSAGVILYILLCGVPPFWAETEQGVAQAIIRSVIDFKRDPWPKVSDNAKDLVKKMLDPDPTRRLSAHHVLDHPWLQNIKKTPNVSLGETVKARLKQFLVMNKLKKKALAVIAEFLSAEEVAGMKEVFEMMDTGKKGKINREELKNGLQKLGHQIPDVDLQILMEAADVDGDGSLNYEEFVAVTIHLRKMANDEHLHKAFSVFDKNQSGYIEIEELRNALSDEDDSNSEDVINAIMHDVDTDKDGRICYQEFAAMMKAGTDWRKASRQYSRERFTSLSLKLMRDGSLQSENKV

>CaCDPK2

MGNTCIGPKLGNNGFLSSVTAAVWKTRQPEPLPLTHKGDSNSHKNQENSSVDSSSKVDGN

GSSDPTSNSFGGTQSTPPPHLKISADTESSEKNANVNKPVEGVKQNNKPSHVKRLSSVGL

KMESVLGRKTENLKEICTLGRKLGQGQFGTTYLCVDMVHGKELACKSIAKRKLSTEEDVEDVRREIQIMHHLAGQPNVVQIVGAYEDAVEVHVVMELCAGGELFDRILQRGHYSEKQAAELARVIVGVVEACHSMGVMHRDLKPENFLFINQDEDSPLKTIDFGLSVFFKPGSEIFTDVVGSPYYVAPEVLRKRYGQECDIWSAGVIIYILLSGVPPFWEETEQGIFEQVLRGELDFVSE

PWPRISESAKDLVRRMLVRDPKKRMTAHEVLCHPWVRVGGVAPDKPLDSAVLSRLNQFSAMNKLKKIAVRVIAESLSAEEIAGLKEMFKMIDTDNSGSITLEELKTGLERVGANLKDSEI

TSLMQAADTDNSGTIDYGEFIAAMLHLNKTQKEDHMYAAFSYFDQDGSGYITKDELQQACDKFGMSNISIEELMREVDQDNDGRIDYNEFVAMMQDTGFGDK

>CaCDPK3

MGNCCSRGEEQPNVVTNNNENGEQINNTKNEENNSSIHNVDMPQGSTTPTKTSPPHTSPS

PSSKPSKQAPIGPVLGRPMEDVRATFTIGKELGRGQFGVTHLCTHKQSGDQFACKTIAKR

KLVNKEDIEDVRREVQIMHHLTGQENIVELKGAYEDKHSVHLIMELCAGGELFDRIITKG

HYTERAAASLLRTIVEIVHTCHSMGVIHRDLKPENFLLLNKDENAPLKATDFGLSVFYKQ

GDIFKDIVGSAYYIAPEVLKRRYGPEVDIWSIGVMLYILLCGVPPFWAENENGIFSAILR

GHVDFSSDPWPSISNGAKDLVRKMLTVDPRQRLTAMQVLNHSWIKEDGEAPDTPLDNVVLHRLKQFRAMNKFKKVALRIIAGCLSEEEIMGLKQMFKNMDTDNSGTITLEELKQGLAKQGTKLSDYEIKQLMEAADADGNGTIDYEEFITATMHMNKMDREEHLYTAFQYFDKDHSGYISREELEQALREFGMDDENDLKEIINEVDTDHDGRINYDEFVAMMKKGNPEAATINPRKRRDSFVA

>CaCDPK4

MITDKKKPRGIKEPPTDRQPESHHSMQNKPPEMVKIEREKPKKEQKKDTQVVKQEKMSVDLKPTEAIELPGKSDNTIHENAKAKQSTKPKKPPHVKRMLSAGLQAEFVLKTKTGLLKEHFELGEKLGHGQFGTTFLCIEKATGNKYACKSIAKRKLLTPEDVEDVRREIQIMHHLSGNPN

VISIKAAYEDSVAVHVVMELCSGGELFDRIVKQGHYSERQAAELARTIVGVVEACHSLGV

LHLDLKPENFLFVNEKEDSPLKIIDFGLSMFFKPGQIFSDIVGSPYYVAPEVLKKCYSQE

ADIWSAGVIIYILLTGVPPFWGESEQEIFDEVLRAKIDFTSDPWPNISEDAKDLVRRMLV

REPKARLTAHEVLCHPWVKIDGVAPDKPLDSAVLSRLTQFSAMNKLKKMALMVIAESLSE

EEIAGLKEMFKMIDTDNSGHITLDELKIGLKQFGADLSETEIRELMKAADVDNSGTIDYG

EFVAAMLHVNKAEKEDYLSAAFSYFDKDGSGYITADELQKACEEFGIKDVHLDEIIQEVD

QDNDGRIDYSEFKAMMQKGNVNFGNRRLPNNFSIGFRDATKVH

>CaCDPK5

MGICVSKSKPNANNGHRGSSGVHSNGPHRTEIQYTKSPGPEAQLPVRPPPSPKPAVRYDT

ILGKPYEDVRLHYTLGKELGRGQFGVTYLCTEIATGQQYACKSISKRKLVTKADKDDMRR

EIKIMQHLSGQPNIVEFKGAYEDKGSVYLVMELCGGGELFDRIIAKGHYSERAAATMCRA

IVNVVHVCNFMGVLHRDLKPENFLLSDKSENAALKITDFGLSVFIQEGKLYKDIVGSAYY

VAPEVLKRCYGKEIDIWSAGVMLYILLSGVPPFWAETEKGIFDAILKGNIDFESEPWPSV

SSSAKDLVQKMLTKDPKKRITAAQVLEHAWLKEGGVASDKPLEGAVLSRMKQFRAMNKLKKLALKVIAENLSAEEIHGLKAMFNNIDTDNSGSITYEELKNGLAKLGSKLTEAEVKQLMEAVRQNSYKLLMFFCSLNY

>CaCDPK6

MGNACRGSFRAKTFQGSYPQNHSTSKHSKVEEEQQPDPAPLVVKDHSTSTKNMNHRSSANQSCCVLGHKTPNIRDLYALGRKLGQGQFGTTYLCTELSTCIDYACKSIAKRKLISKEDVE

DVRREIQIMHHLAGHKNIVSIKGAYEDPLYVHIVMELCGGGELFDRIIQRGHYTERKAAD

LTKIIVGVVEACHSLGVMHRDLKPENFLLVNKDDDFSLKAIDFGLSVFFKPATLLIKYDL

GDAFSHYPSFLEILAAANMDCQIFTDVVGSPYYVAPEVLLKNYGPEADVWTAGVILYILL

SGVPPFWAETQQGIFDAVLKGHIDFDSDPWPLISESAKDLIRKMLCMRPSERLTAHEVLC

HPWICENGVAPDRALDPAVLSRLKHFSAMNKLKKMALRVIAESLSEEEIAGLKEMFKAMD

TDNSGAITFDELKAGLRKYGSTLKDIEIRELMDAADVDNSGTIDYGEFIAATIHLNKLDR

EEHLMAAFQYFDKDGSGYITVDELQQACADHNITDVLFEDIIREVDQDNDGRIDYGEFVA

MMQKGNPCIGRRTMRNSLNFSMRDAPGAH

>CaCDPK7

MGGCFSRNKYAQRDGKGYRPRPRKGSSSAYHHYKHPVYYHEAYPRPPQERPSYKPLPQPQQQQKQKQAHQVTVKITQPRIQVIQRIGQDAILGKPFEDIKAHYTLGRELGRGQYGVIYLC

TENSTRQHYACKSVLKRKLVCRKDREDIKREIQIMQHLSGQSNIVEFKGAYEDNYSVHLL

MELCAGGELFDRIIAKGYYSEKDAADIIRQIVNVVYICHFMGVMHRDLKPENFLLTSKDE

HVMLKATDFGLSVFIEEGKVYRDIVGSAYYVAPEVLRRSYGKEADVWSAGVILYILLSGV

PPFWDGKDRDENIIRCCTGCYFLHFSFNLSCQIFSETEKGIFHAILKGEIDFQSDPWPSI

SNCAKDLVRKMLTQDAKNRITSGEVLEHPWLQSGEASDKPIDSAVLSRMKQFRAMNKLKKLALKVIAENLSEEEIKGLKSMFANMDTDNSGTITYEELKSGLARLGSKLSEAEVKQLMEAADVDGNGTIDYIEFVTATMHRHRLERDDHLFKAFQYFDADHSG

>CaCDPK8

MGNICSASSKVSGSNSNTPFTTTTTVNGHRNRKNSSNTSRKQEGSHCNKQKAKDKQQTKNAQKNVKHNSRKQSGVIPCGKRTDFGYDKDFDNKFTIGKLLGHGQFGYTYVATDKSNGNRVAVKRIEKKKMVAPIAVEDVKREVKILKALAGHENVVEFYNAFEDDNYVYIVMELCEGGELLDRILAKKDSRYTEKDAAIVVCQMLKVAAQCHLHGLVHRDMKPEVCKKLCTSFGLLMLLHNFLFKSPKEDSELKATDFGLSDFIRPGKKFQDIVGSAYYVAPEVLKRRSGPESDVWSIGVITFILLCGRRPFWDKTEDGIFKEVLRNKPDFRRKPWPTISNSAKDFVKKLLVKDPRARLTAAQALSHPWVREGGDASEIPLDISVLSNMRQFVKYSRLKQFALRALASTLDEEELADARDQFSAIDVDKNGVISLEEMRQALAKDLPWKMKESRVLEILQAIDSNTDGLVDFPEFVAATL

HVHQLEEHNSIKWQQRSQAAFEKFDIDRDGFITPEELRMHTGLKGSIAPLLEEADVDKDG

KISLSEFRRLLRTASISSRMVNSPRFRGSRKS

>CaCDPK9

MGNCNACIRPEEASKTDPSPTKPKKPRERRPNPYSGSPAPIRVLKDFFPKTRISDKYILG

RELGRGEFGVTYLCTDRETREALACKSISKKKLRTAVDIEDVRREVAIMSSLPDHPNIVK

LRATYEDNEAVHLVMELCEGGELFDRIVARGHYSERAAAGVARTVAEVVRMCHANGVMHRDLKPENFLFANKKEHSALKAIDFGLSVFFKPGERFSEIVGSPYYMAPEVLKRNYGPEVDIWSAGVILYILLCGVPPFWAETEQGVALAILRGVIDFKREPWPQVSEKAKSLVRQMLEPDP

KKRLTAQQVLDHPWIQNAKKASNVPLGDIVRTRLKQFSIMNRFKKKALRVIAEHLKLEEI

EVIREMFALMDCDGDGKITYDELKAGLRKVGSQLAEAEMKLLMDVADVDGNGVLDYGEFVAVIIHLQRMENDEHFRRAFMFFDRDGSGYIELDELREALADDSGACDTDVVNEIMREVDTDKDGQISYEEFVAMMKTGTDWRKASRQYSRERFKSLSVNLMKDGSLQLQDVLSGQTVIV

>CaCDPK10

MGICLSKTKPAESKSHGHHRSGGGGGGGGHHHHRGTHQTQIQYTKSPGQEAQLPTRPQASPKPVFKQETILGKPFEDVKAHYTLGKELGRGQFGVTFLCTEIETGHQYACKSISKKKLVT

KSDKADMRREIQIMQHLSGQPNIVAFKGAYEDKSSVCLVMELCAGGELFDRIIAKGHYSE

SAAASMCRAIVNVVHVCHFMGVMHRDLKPENFLLLDKSENAALKATDFGLSVFIEEGKVYKDIVGSAYYVAPEVLRKSYGKEIDVWSAGVMLYILLSGVPPFWAGKDFLFLLVSGFCNAETERGIFDAILKEDIDFESQPWPSISSSAKDLVRKMLNKDPKERISAAQVLEHPWLKVGGV

ASDKPLDNAVLSRMKQFRAMNKLKRLALKVIAENLSADEIQGLKSMFHNIDTDNSGTITY

EELKSGLARLGSKLTEAEVMQLMEAADVDGNGSIDYIEFITATMHKHRLEREENLYKAFQ

YFDKDGSGFITRDELETSMEEHGIGDPASIREIISEVDADNDGRINYEEFCTMMRSGAKQ

PGKLF

>CaCDPK11

MGLCFSKACCCPNYKDVPISSSPSASPEYHPVPILSQKYPEESPPAPISSYMPMPRTSTS

SSSSTNQFGQILGKPYTDIKTIYDLDKELGRGQFGITYLCTEKATGLKYACKSISRRKLV

TQKDIEDVRREVTILQYLSGQPNIVEFKGAYEDKNNLHLVMELCSGGELFDRITVKGNYS

EKEAARIGRQIVNVVHACHFMGVMHRDLKPENFLMVSKDDDSPLKATDFGLSVFIEEGKI

YKDIVGSAYYVAPEVLKRNYGKEIDVWSAGVILYILLSGFPPFWAETEKGIFEEIVKGKL

DFESSPWPSISSSAKDLVRKMLTMDPRRRITADEALGHPWLKKDGEASDKPIDSAVLLRL

KQFRAANKMKKLALKVIAENLSEEEIKGLKQMFNNMDTDGSGTITYEELKTGLSRLGSKLTEAEIKQLMEAADVDNSGTIDYIEFITATMHRHKLEREENLYKAFRFFDKDNSGYVFITR

DELRHAMEEYGMGDEATIDEILDDVDTNKDGLINYDEFVAMMRRGTTDHEAKQIR

>CaCDPK12

MEIPNAENSTTQKKLPTVSSTKESLNVLPYQTPRIGEYYTLGKKLGQGQFGTTYLCSENA

TGLEYACKSIPKRKLFCKEDYEDVWREIQIMHHLSEHPYVVRIKGTYEDNVFVHIVMEVC

KGGELFDRIVQKGHFTEKKAAQLVKTIVKVVEACHSLGVMHRDLKPENFLFDSSDEDAKLKATDFGLSIFYKPGQYFSDVVGSPYYVAPEVLHKYYGPEIDVWSAGVILYILLSGVPPFW

AGKLCTSDYFIASFFTDNGIFKQILKGKIDFESEPWPQISDSAKDLVKKMLTRDPRARLT

AHQVLCHPWIVDDNVAPDRPLGSAVLSRLKQFYDMNKFKKMALRVIAERLSEEEIGGLRQ

LFKMIDTDNSGTITYEELKHGLKRVGSDLTEAEIKALMSAADFDNNGTIDYGEFIAATLH

LNKMEREENLLAAFSYFDKDGSGYITIDELQQAFQEFGLGDVKLEEIIKEIDIDNDGRID

YGEFATMMKKGNTGPGARTMRGNLTFSIADALGSQ

>CaCDPK13

MGSCFSSSKVSGSNSNTPSATNTNTNITNTTSASGTVPTNPRETSKAATSTTAVNSRKQE

GSNYNQKKHHQQQQKQQPRNSQQNVKTISSRRQSGVIPCGKRTDFGYDKDFEKRYTIGKLLGHGQFGYTYVATDKSSGDRVAVKRIEKNKMVLPIAVEDVKREVKILKALAGHENVVQFYNSFEDENYVYIVMELCEGGELLDRILSKKDSRYTEKDAAIVVRQMLKVAAECHLHGLVHRDMKPENFLFKSSKADSPLKATDFGLSDFIRPVLWFSLVKLITYRRKLIYAGKKFQDIVGS

AYYVAPEVLKRRSGPESDVWSIGVITYILLCGRRPFWDKTEDGIFKEVLRNKPDFRRKPW

SNISNSAKDFVKKLLVKDPRARLTAAQALSHPWVREGGDASEIPLDISVLSNMRQFVKYS

RLKQFALRALASTLDEEELADLRDQFSAIDVDKNGVISLEEMRQALAKDLPWKMKESRVLEILQAIDSNTDGLVDFPEFVAATLHVHQLEEHNSTKWQQRSQAAFEKFDVDKDGFITPEE

LKMHTGLRGSVDPLLEEADIDKDGKISISEFRRLLRTASMSSPTVRDSRRNAAL

>CaCDPK14

MGGCFSKKYTQQDANRYGAGRRSGNQEYQKSPQPRSERSYQPQPQPTYQPQPPPERPYQQQSQTKPQQQAHPVPVTVPPPAQPQDQIQGPHLTDILGKPFEDIRKLYTLGKELGRGQFGV

TYHCIENSTGNAYACKSILKRKLVSKNDREDMKREIQIMQHLSGQPNIVEFKGAYEDRHS

VHLVMELCAGGELFDRIIARGYFSEKDAAEIIRQIVNVVNICHFMGVMHRDLKPENFLLS

SKDENAMLKATDFGLSVFIEEGKVYRDIVGSAYYVAPEVLRRSYGKEADVWSAGVILYIL

LSGVPPFWAETEKGIFNAILKGEIDFQSDPWPSISHSVKDLIRKMLTQEPKKRITSAQVL

EHPWLRLGEASDKPIDSAVLSRMKQFRAMNKLKKLALKVIAENLSEEEIKGLKAMFDNID

TDNSGTITYEELKSGLARLGSKLTETEVKQLMEAADVDGNGTIDYIEFITATMHRHRLER

DEHLFKAFQHFDKDNSGFITMDELEHAMKEYGMGDESTIKEIIAEVDTDNDGRINYEEFC

AMMRSGTTQPQQKLF

>CaCDPK15

MTMPKSEQKEVEPVKKEEVDKEERPVKPKKLVEMKRVGSAGLRTDSVLQKKTGNLKEFFSIGKKLGQGQFGTTFKCIEKGTRKEYACKSIAKRKLLTDDDVEDVRREIQIMHHLAGHPNVISIKGAYEDAVAVHVVMEHCAGGELFDRIIQRGHYTERKAAELTRTIFGVVEACHSLSVM

HRDLKPENFLFVDQKEDSLLKTIDFGLSVFFKPGERFTDVVGSPYYVAPEVLKKRYGPEA

DVWSAGVIVYILLSGVPPFWAENEQGIFEQVLHGDLDFSSDPWPSISEDAKDLVRGMLVR

DPRKRLTAHEVLCHRWVQVDGVAPDKPLDSAVLSRMKQFSAMNKLKKMALRVIAESLSEEEIAGLKEMFKMIDTDNSGQITFEELKVGLKRVGANLKESEIYDLMQAADVDNSGTIDYGEFIAATLHFNKIEREDHLFAAFSYFDKDGSGYITADELQQACEEFGIGDAHLEDMIRDADQ

DNDGRIDYNEFVAMMQKGHPVTVGGKKGLEHSFSTGFRDALKV

>CaCDPK16

MKSTAIDQESCSKNTSRTSVPKSQQFSRPMSVVKDPTGNDIYQRYEFGKELGRGEFGITY

RCVDKVSGENVACKTIAKSKLRTEIDVEDVKREVIIMRHLPKHPNIVSYKEVYEDKEAVY

LVMELCEGGELFDRIVARGHYTERAAALVTKTILEVVQVCHKHGVIHRDLKPENFLYANA

TENAQLKAIDFGLSIFFEPGKSLGFKSSHPILYTILFGEIVGSPYYMAPEVLRRNYGQEV

DVWSAGVILYILLCGVPPFWAGNLQLKVLVISFTETEEGIAHAIVKGTIDFNRDPWPRVS

DEAKDLVKGMLEANPYNRFTVEEVLEHHWIQNADKVSNTLLGEGVRAKIKQFTLMNKFKKKVLRVVADNLPQDQVHGIKQMFYMMDTDHNGNLGFQELKDGLHMMGQNVAEPDVKVLMDAADVDGNGMLNCEEFVTMAVHLQRLSNDDLLRQAFLQFDKNKSGFIEFEDLKISLFDDHHAPQNDQVINDIIFDADLDKDGRISYQDFKVMMSTGTDWKMGSRQYSKAMLNALSMRLFKDKSMQLKN

>CaCDPK17

MAQVAAKKRPPISSKPSPNVLPYQTPRLREHYSLGNKLGQGQFGTTYQCTEKETGLQYAC

KSIPKRKLLCREDYEDVWREIQIMHHLSEHTNVVRIKGTYEDNLFVHIVMELCKGGELFD

RIVQKGHYTERKAAHLMKTIVKVVEACHSLGVMHRDLKPENFLFDSSDEDATLKATDFGLSIFYRPGQYISDVVGSPYYVAPEVLHRFYGPEIDVWSAGVILYILLSGVPPFWAETDNGI

FKQILKGKMDFESEPWPHISDNAKDLVKKMLNRDPKARITAHEVLCHPWLVDDAAAPDKPLGSAVLNRLKQFYDMNKLKKMALRVIAERLSEEEIGGLKQLFKMIDTDNSGTITYEEMKEGLKRVGSDLVESDIRALMKAADLDNNGTIDYAEFIAATLHLNKMEREENLLAAFSYFDKDGSGYITTDELQQACIEFGLGDVKLDDLIKEIDIDNDGRIDYGEFATMMKKGNTGFAARTMRGNLNFNLADALGASDSEKNQ

>CaCDPK18

MGNCCRSPAAVAREDVKSSNYSGHDHLRKEKSINKQKQQINVLTDKKNENVEEKYIIDRE

LGRGEFGVTYLCIDRKNRDLLACKSISKRKLRTAVDVEDVRREVAIMKHLPMDSSIVSLK

EACEDDNAVHLVMELCEGGELFDRIVARGHYTERAAVAVTRTIMEVVEVCHKHGVIHRDL

KPENFLYANKKENSPLKAIDFGLSIFFKPGEKFSEIVGSPYYMAPEVLKRNYGPEIDIWS

AGVILYILLCGVPPFWAESEQGVAQAILRGAIDFKREPWPSISDSAKNLVRQMLEPDPKL

RLTAKQVLEHPWLQNAKKAPNVPLGDMVKSRLKQFAMMNRFKRKALRVIADFLSNEEVEDLKEMFSKIDTDNDGIVSVEELKAGLQKVNSQLADSEIKMLIEAIDTNGKGTLDYGEFIAISLHLQRMSNDEHLHRAFSFFDKDGNGYIEPDELRDALMEDGSDDCTNVANDIFQEVDTDKDGRISYDEFAAMMKTGTDWRKASRHYSRGRFNSLSVKLMKDGSLNLG

>CaCDPK19

MDSSKPKTSSLSTPSKLFWVLPYKTQSLESLYTLGKKLGQGQFGTTYL

CTEKSTSNLYACKTIPKKKLICKEDYEDVWREIQIMHHLSEHPNVVRIKG

TYEDALCVHIVMELCAGGELFDRIVQKGQYSEKEAAQLIKTIVGVVEACH

SLGVMHRDLKPENFLFLSSQEDAALKATDFGLSVFYKPGETFSDVVGSPY

YVAPEVLCKHYGPESDVWSAGVILYILLSGVPPFWAETDMGIFRQILRGK

LDFESEPWPGISDSAKDLIRKILDRNPKRRLTAHEVLCHPWIVDDSMTPD

KPLDSAVLSRLKQFSAMNKLKKMALRVIAERLSEEEIGGLKELFKMLDTD

NSGTITFEELKEGLRRVGSELMESEIKDLMDAVRIILVVFCVLEIYIDNS

GTIDYGEFIAATVHLNKLEREENLLSAFSYFDKDGSGYITIEELQQACQE

LGLSELNLDEIIKDIDQDN

>CaCDPK20

MANHAEEVKQNKPTHVRRKSSIGLHVESVLGRKTGNLKDVFSLGRKLGQGQFGTTFLCLEKSPCGMVCACKSIAKRRLTTEEDIEDVRREIQIMHHFAGQPSVVQIIGAYEDAVAVHVVM

ELCAGGELFDRILQRGHYSEKKAAELARVIISVVEACHSLGVMHRDLKPENFLFVNQQEE

SPLKAIDFGLSVFFKPGIRYCFDVVGSPYYVAPEVLRKHYGLECDIWSAGVIIYILLSGG

PPFWDEMEQGIFEQVLTGELDFASEPWPSISESAKDLVRKMLVRDPKKRLTAHEVLCHPW

VRVGGVAPDKPLDCAVLSRLNQFSAMNKLKKIAVRVIAESLSGEEIAGLKEMFKMIDADN

SGQITLEELKTGLERVGANVEDSEIVSLMQAADIDNSGTIDYGEFIAAMLHLNKIEKEDH

MYAAFSYFDEDGSGYITQDELQKACDKFGISNIPIDELMREVDQDNDGRIDYSEFVAMMQ

DTGFGNK

>CaCDPK21

MGNNCVGPKLANNGFLQSVSAAVWKPNQSENLPLPNEGEGESKSEKNKENLVDSSKTDGSCKQGNPPPDHMINGEVDKNNKNDVLTANHAEKVKQNKPTHVRRKSSIGLHVESVLGRKTGNLKDVFSLGRKLGQGQFGTTFLCLEKSPGGMVCACKSIAKRRLTTEEDIEDVRREIQIMHHLAGQPSVVQIIGAYEDAVAVHVVMELCAGGELFDRILQRGHYSEKKAAELARVIVSVVEACHSLGVMHRDLKPENFLFVNQQEESPLKAIDFGLSVFFKPGEIFSDVVGSPYYVAPEVL

RKHYGLECDIWSAGVIIYILLSGVPPFWDETEQGIFEQVLTGELDFASEPWPSISESAKD

LVRKMLVRDPKKRLTAHEVLCHPWVRVGGVAPDKPLDCAVLSRLNQFSAMNKLKKIAVRVIAESLSGEEIAGLKEMFKMIDADNSGQITLEELKTGLERVGANVEDSEIVSLMQAANIDN

SGTIDYGEFIAAMLHLNKIEKEDHMYAAFSYFDEDGSGYITQDELQKACDKFGISNIPID

ELMREVDQDNDGRIDYSEFVAMMQDTGFGNKGSK

>CaCDPK22

MGNNCVHEKISKDGFFSSLWWSRSPDMIMYEKKESSSQASTDSIPNKPPELVKIDSNPSD

VKGAEQVIIIVTDEKKDARVVKPEDMIKISVDLKPVQITGPEKAKPAEPAKPRKPHNVKR

MASAGLQVDSVLKTKTGHLKEHYNLGDKLGHGQFGTTFLCIEKGTGKKYACKSIAKRKLLTDEDVDDVRREIQIMHHLSGNPNVISIKGAYEDPVAVHVVMELCTGGELFDRIIKRGHYS

ERQAAELARTIVGVVEACHSLGVMHRDLKPENFLFVNDEENSPLKTIDFGLSMFFKPGQI

FDDVVGSPYYVAPEVLRKRYGPEADIWSAGVIIYILLSGVPPFWGESEEEIFDEVLHGDI

DFELNPWPKISQGAKDLVRRMLVKDPKKRLTAHEVLCHPWVQIDGVAPDKPLDSAIFTRL

TQFSAMNKLKKMAIRVIVERLSEEEIAGLKEMFKMIDTDNSGQITFDELKIGLKKFGANL

NESQIHDLMKAVSNRHARCELFRLANI

>CaCDPK23

MGTCNSTLSSDQTTAGAAASPPSTTGIRILPSTQPPPPRPLSGVGRILNRPLEDIRSTYI

FGGELGRGQFGVTYLVTHEKTRLQYACKSIATRKLINKDDVDDVRREVQIMHHLTGHRNI

VELKGTFEDMHHVHLVMELCAGGELFDRIIAKGHYSERAAAGVCREIVTVVHNCHSMGVMHRDLKPENFLFLSSDESSPLKATDFGLSVFFKPGDVFKDLVGSAYYVAPEVLRREYGPEA

DIWSAGVILYILLSGVPPFYGENDQSIFDAVLRGHLDFSSDPWPSVSSSAKDLVKKMLRS

DPRERISAAEVLNHPWMREDGDASDKPLDIAVLSRMKQFRAMNKLKKVALKVIAENLSEEEIIGLKEMFKSIDTDNSGTITFEELKAGLTKMGTKLSESEVRQLMEAADVDGNGTIDYLE

FITATMHMNRMEREDHLYKAFEYFDKDKSGYITMEELEHALKEYNITDEKTIKEIIAEVD

TDNDGRINYDEFAAMMRKGNPDFVNNRRRR

>CaCDPK24

MGNCCAVPKTSDTEEKKRGKNKPNPFSVDYAHGNGHKSYVLDNPTGCDIEATYELGRELGRGEFGVTYLSTDKATGDVYACKSISKKKLRTRVDIEDVRREVEIMKHLPKHPNIVTLKDT

YEDDNAVHIVMELCEGGELFDRIVARGHYTERAAAAVTRTIVEVIQMCHKHGVMHRDLKPENFLFENKKETAPLKAIDFGLSVFFKPACHVGFIGERFNEIVGSPYYMAPEVLKRDYGPE

VDVWSAGVILYILLCGVPPFWAETEQGVAQAIIRSVVNFKRDPWPKVSDNAKDLVKRMLNPDPSKRLTAQEVLDHPWIQNAKKNPNVSLGETVKARLKQFSMMNKLKKRALRVIAEHLTVDEVAGIKEGFQLMDIGNKGKIDINELRVGLQKLGHQIPESDVQILMDVGDVDKDGFLDYGEFVAISVHLRKMANEEHLKAAFEFFDKNQNGYIEIDELREALDDEIETNSEEVINAIMQDVDTDKDGRISYDEFSAMMKAGTDWRKASRQYSRERYNSLSLKLMKDGSLQS

>CaCDPK25

MGNCCVTPDNNSSEKKKNKKKNKPNPFALDYGGTRASSEGDGNKLIVLKDPTGHNIQEKYDLGHELGRGEFGVTYLCTDNDTGEKYACKSISKKKLRTAVDIDDVRREVEIMKHLPKHPNIVSLKDTYEDDNAVHIVMELCEGGELFDRIVARGHYTERAAAGIMKTIVEVVQMCHMHGVMHRDLKPENFLFSNKKETAPLKAIDFGLSVFFKPGEHFNEIVGSPYYMAPEVLKRNYGPEVDVWSAGVILYILLCGVPPFWAETEQEVAQAIIRSVVDFKRDPWPKVSDNAKDLVKKMLDPDPTQRLTAQQVLEHTWLQNIKKAPNVSLGETVKARLKQFSVMNKLKKRALTIIAEFLSAEEVAGMKDAFDMMDTGKKGKINLGELKNGLQKLGHQIPDADLQILMEAADVDGDGSLNYGEFVAVSVHLRKMANDEHLHKAFSVFDRNQSGYIEIEELRSALSDEDGGNSEEVINAIMHDVDTDKDGRISYEEFAAMMKAGTDWRKASRQYSRERFNSLSLKLMRDGSLQVGK

>CaCDPK26

MQGSTTPSKSPPPHASPNHSSKPSKAAPIGPVLGRPMEDIKATYTLGKELGRGQFGITHL

CTHKQTGEQFACKTIAKRKLVNKEDIEDVRREVQIMHHLTEQPNIVELKGAYEDKHSVHL

VMELCAGGELFDRIIARGHYTERAAASLLRTIVQIVHTCHSMGVIHRDLKPENFLLLSKD

EDAPLKATDFGLSVFYKQGDEFKDIVGSAYYIAPEVLKRKYGPEVDIWSIGVMLYILLCG

VPPFWAESENGIFNAILRGHVDFSTDPWPSISSGAKDLVKKMLNSDPSQRLTAVQVLNHP

WIKEDGEAPDVPLDNAVLNKLKNFRAMNKFKKVALRVIAGCLSEEEIMGLKQMFKGIDTDNSGTITLEELKQGLARQGNKLSDYEIKQLMESADADGNGTIDYEEFITATMHMNRMDREDHLYKAFQYFDKDSSGYITMEELEQALREFGMNDTKDIKEIISEVDSDNDGRINYDEFVAMMKKGNPEIATNVKKRREVLVN

>CaCDPK27

MGNYCCSRGQPNDGDHHMSTMTETSNTKNDQSIANKSKKQKHAPVTPPKSPDPSSKPSKNSPIGPVLGRPMEDVRKTYSIGKELGRGQFGVTHLCTHKQSGEQFACKTIAKRKLVNKEDI

EDVKREVQIMHHLTGQQNIVELKGAYEDKNSVHLVMELCAGGELFDRIIAKGHYTERAAATLLRTIVQIVHTCHSMGVIHRDLKPENFLLLNKDEDSPLKATDFGLSVFYKQGDVFKDIV

GSAYYIAPEVLKRRYGPEVDIWSVGVMLYILLSGVPPFWAETEHGIFNAILRGHVDFSSD

PWPSISHGAKDIVRKMLTTDPKQRLTAIQVLNHPWIKEDGDAPDTPLDNAVLSRLKQFRA

MNNFKKVALRVIAGCLSEEEIMGLKQMFKSMDADNSGAITLEELKQGFAQQGTKLSDYEI

QQLMEAADADGNGTIDYEEFITATMHMNRMDKEEHLYTAFQYFDKDNSGYITLEELEQALLEFGIDDGKDIKEIVAEVDSNNDGRINYEEFAAMMRKGTPDAAAELKKRRESFVA

>CaCDPK28

MWGSRSPDDTASTITNGESVRIETPTSVKETDSNPLPVQDKPPEHITMPKSEPKEEEKPK

KPEKPAEMKRVASAGLRTDSVLQKKTGNLKEFFSIGKKLGQGQFGTTFLCTEKATGKRYA

CKSIAKRKLLTDDDVEDVRREVQIMHHLAGHPHVISIKGAYEDAVAVHLVMEYCAGGELF

DRIIQRGHYTERKAAELTRTIVGVVEACHSLGVMHRDLKPENFLFVDQKEESLLKTIDFG

LSIFFKPGSDKFTDVVGSPYYVAPEVLRKRYGPEADVWSAGVIIYILLSGVPPFWSENEQ

GIFEQVLHGDLDFTSDPWPSISEGAKDLMRRMLIRDPRKRLTAHEVLCHPWVQIDGVAPD

KPLDSAVLSRMKQFSAMNKLKKMALRVIAESLSEEEIAGLKEMFRMIDTDNSGQITFEEL

KDGLKRFGSNLKESEIYDLMQAADVDNSGTIDYGEFIAATLHLNKIERQDHLFAAFSYFD

KDGSGYITADELQHACEEFGIGDVRMEEMIREADQDNDGRIDYNEFVAMMQKGNPVVGGGKKGLEHSFSIGFREALKL

>CaCDPK29

MGNCCRSPAAVAREDVKSSNYSANDHNGRREKFNTRTNQKPITVLTDVKK

ENIEERYLVDRELGRGEFGITYLCIDRSRKELLACKSISKRKLRTAVDVE

DVRREVAIMKHLPQNSSIVSFREACEDENAVHLVMELCEGGELFDRIVAR

GHYTERAAANVVRTIVEVVQLCHKHGVIHRDLKPENFLFANKKENSPLKA

IDFGLSIFFKPGEKFSEIVGSPYYMAPEVLKRNYGPEIDIWSAGVILYIL

LCGVPPFWAESEQGVAQAILRGAIDFKREPWPSISEGAKDLVRQMLEPDP

KLRLTAKQVLEHPWLQNAKKAPNVPLGDVVKSRLKQFSLMNRFKRKALRV

IADFLSSEEVEDLKETFNKIDTDNDGVVSVEELKAGLQKLNSQLAESEVQ

MLIEAIDTNGKGTLDYGEFIAVSLHLQRMANDEHLHKAFSYFDKDGNGYI

EPDELRDALMEDGVDDCTNVANDIFQEVDTDKDGRISFEEFAAMMKTGTD

WRKASRHYSRGRFNSLSVKLMKDGSLNLG

>CaCDPK30

MGNTCRGSIGGKTFQGYNQPEDSSCSTNQTNSYTSSDIFSPTSQKNKNNQDNEQSLSLVS

PRKASMNRTSSNQAYYVMGHKTANIRDLYTLGRKLGQGQFGTTYLCTENSSGAEYACKSI

SKRKLISKEDVDDVRREIQIMHHLSGHKNIVTIKGAYEDPLYVHIVMEICSGGELFDRII

QRGHYSERKAAELTRIIVGVVEACHSLGVMHRDLKPENFLLVNKDDDFSLKAIDFGLSVF

FKPGQIFTDVVGSPYYVAPEVLLKHYGPEADVWTAGVILYILLSGVPPFWAETQQGIFDA

VLKGHIDFDSDPWPLISESAKDLIRKMLCMQPSERLTAHEVLCHPWICENGVAPDKALDP

AVLSRLKQFSAMNKLKKMALRVIAESLSEEEIAGLREMFKAMDTDSSGAITFDELKAGLR

KYGSTLKDTEIRELMDAADVDNSGTIDYGEFIAATVHLNKLEREEHLMAAFQYFDKDGSGYITVDEVQQACIEHKMTDVYFEDIIREVDQDNVSPLF

>CaCDPK31

MGNTCSGPTLNKDSADSSKTEGKGSDSDNVQKTPPPHLQIPGDQPLQGDEKSRNDPPKGD

GDGDGDGNDGTVNTNKAVKDVKRSMSRSLKRVMSVGLQVESVLGRKTGNLKDIYSLGRKLGQGQFGTTFLCVDKAQGKEYACKSIAKRKLTTEEDVEDVRREIQIMHHLAGNPSVVQIVGAYEDAVAVHLVMELCAGGELFDRIVNRGHYSEKKAAQLARVIVGVVEACHSLGVMHRDLKPENFLFVNEEEDSSLKTIDFGLSVFFRPGETFTDVVGSPYYVAPEVLRKRYGPECDIWSAGIIIYILLCGVPPFWDETEQGIFEQIVKGELDLVSEPWPAISESAKDLVRKMLVRDPKKRLTAHEVLCHPWVRVGGVAPDKPLDSAVLSRLNQFSAMNKLKKIAIRVIAENLSGEEIAGL

KQMFKMIDADNSGHITLEELKKGLEKVGSHLKDSEINILMQAADMDNSGTIDYGEFIAAM

LHLNKVQKEDHMYAAFSYFDQDGSGYITQEELQQACDKFGLTNIPIEELMREVDQDNDGRIDYNEFVAMMQDNGFGKNGTR

>CaCRK1

MGACTSKPPRPNPYSPQEIFPPLKTPKIDEPHKDNEAKKSPFFPFYSPSPARFFLSKKSP

ARHSSASKSANSTPARLFKRPFPPPSPAKHIKALLLRRHGSVKPNAAAIPEGEESEGANL

DKSFGFNKQFNTKYEIGEEVGRGHFGYTCSAIVKKGELKGQKVAVKVIPKAKMTTAISIE

DVRREVKILRALTGHTNLIQFYDAFEDRDNVYIVMELCQGGELLDRILARGGKYSEEDAK

DVMVQILNVVAFCHLQGVVHRDLKPENFLFSSNDESSQLKAIDFGLSDYVRPDEKLNDIV

GSAYYVAPEVLHRSYGIEADVWSIGVIAYILLCGSRPFWARTESGIFRAVLKADPTYDEA

PWPTLTSEAKDFVKRLLNKDPRKRMSAAQALCHPWMRNHSGTKLPLDILIFRLMKTYMRSSSLRKAALRALSKTLTADELFYLKGQFALLEPDKNGSVKLENIRSALLKYGTDAMKESRI

PDFLASLNALQYRKMDFEEFCAAALSVHQLEALERWEQHARCAYEIFDKDGNKAIVIEEL

ASELGLGPSIPVHAVLHDWIRHTDGKLSFLGFVKLLHGPSTRGLAKAQ

>CaCRK2

MGGCTSKPPPEPHYSKSDLYTPHVTANNDENTSKEEPQVGKKSPFFPFYSPSPAHYFFSK

KSPANSTPRRFFPPPSPAKHIRALLARRHGTVKPNTIPEGDELEGGGGLDKSFGFSKNFG

NKYEIGEEVGRGHFGYTCKAKFKKGELKGQEVAVKVIPKAKMTTAIAIEDVRREVKILRA

LTGHDNLVKFYDAYEDHENVYIVMELCEGGELLDRILSRGGKYSEDDARTVLVQILKVVA

FCHLQGVVHRDLKPENFLFMSKDENAQLKAIDFGLSDFVKPDERLNDIVGSAYYVAPEVL

HRSYSTEADVWSIGVIAYILLCGSRPFWARTESGIFRAVLKADPNFEEQPWPTLSSEAKD

FVKRLLTKDPRKRMTAAQALGHPWIKNSHDIEVPLDIMIFKLMKTYMRSSALRKAALRAL

SKTLTIDELVHLKQQFSLLEPNKNGTINVDNIKAALMKNATDAMKVARIHDFVASLNALQ

YRRMDFEEFCAAALSVYQLEALDKWEQHARCAYEIFEKDGNRAIVIEELASEFGLGPSVP

VHAVLLDWIRHTDGKLSFLGFAKLLHGVSSRAITKIQ

>CaCRK3

MGACTSKPSNYSGDNIAVAVNGATLPVKSTPNNNEEQDRNKKDELNVGKKSPFFPFYSPS

PAHYLFSKKSPANASSNSTPRRFFKRPFPPPSPAKHIRAVLARRHGTVKPNESVIPEVNE

IEASGDGDCGAGLDKSFGFSKNFVSKYELGEEVGRGHFGYTCKAKFKKGEVKGREVAVKVIPKTKMTTAIAIEDVRREVKILRALTGHNHLVKFYDSYEDYNNVYIVMELCEGGELLDRI

LSRGGKYAEDDAKDVMIQILKVVAFCHLQGVVHRDLKPENFLFTSKEETAQLKAIDFGLS

DFVKPDERLNDIVGSAYYVAPEVLHRSYSTEADVWSIGVIAYILLCGSRPFWARTESGIF

RSVLKADPCFEEQPWPTLSSEAKDFVKRLLNKDPRKRMTAAQALGHPWIKNSHNVEVPLDILIFKLMKAYMKSSALRKAALRALSKTLTVDELFYLKEQFALLEPSKNGTISFDHVKTAL

MKHATDAMKEARIHDFLASLNALQYRRMDFEEFCAAALSVHQLEALDRWEQHARCAYEIFEKEGNRAIMIEELASELGLGPSVPVHAVLHDWLRHTDGKLSFLGFAKLLHGVSSRSITKVQ

>CaCRK4

MGQCCSKGVSGNNDGSVVAVTDGNAAVSGNHRPKPPRSPVAHQSVGNGTNYTNNSTPAHSFTTSPFQSPYPAGIAPSPSPVGTPRRKFKWPFPPPSPAKPILSAILKRQGNTSVKPKEGP

IPEDEGGEGERQLDKSFGYPKNLTSKYDLGKEVGRGHFGHTCMAKGKKGELKNQPVAVKIISKAKMTTAISIEDVRREVKILKALSGHQNLVKFYDAFEDVNNVYIVMELCEGGELLDRI

LSRGGRYTEEDAKSIVVQILNVVAFCHLQGVVHRDLKPENFLFAKKDEDSLMKVIDFGLS

DFIRPEQRLNDIVGSAYYVAPEVLHRSYSIEADMWSIGVITYILLCGSRPFWARTESGIF

RSVLRADPNFEDSPWPAVSAEARDFVKRLLNKDHRKRMTASQALAHPWLRTENPSVPLDI

LIFKLVKSYIRTSSLKRAALKALSKALTEEELIYLRAQFNLLEPKAGRLSLDNFRMALMK

QTTDAMREARVLEILNLLEPLSYQQMEFEEFCAAAISTYQLEALENWEQIASVAFHYFEQ

EGNRCISVEELAREMNLGPTAYTFLKDCIRPSDRKLSFLGYTKFLHGVTVRGSSTRHHR

>CaCRK5

MAVAISNSNTETSLYTCCCYKVANLSETILDANYTANLHDRYVLGEQLGWGQFGIIRTCS

DKFTGEVLACKSIAKNRLVTQEDVRSVKLEIEIMTRLSGHPNVVDLKAVYEEEDNVHLVM

ELCAGGELFHQLERHGRFSEAEARVLFHDLMEVVMYCHHKGIVHRDLKPENILLATKGSS

SPIKLADFGLATYIRPGQSLHGTVGSPFYIAPEVLAGGYNQAADIWSAGVILYILLSGIP

PFWGKTKSKIFDAVRAADLRFPSDRWETISSSAKELIKGMLCTDPSQRLTTQQILDHAWV

RDSLPHFNVPRLKVHSDEGSCCSSLMARNQDISFGTCSAVLIDVQSPRFTCKTSFSTLLT

EQSTTSYGSAVFSFSSAGGSNGHDFASPVLTLPSFTFFGPNLVVDQGSYEVDLSVRASDV

DLLHTDASVGKVLMLSDSPVSFEQVVRDKTAEVRKSGSNGSRTLGIQSRRNHTIGLGEFE

QIDIVVTESVIRWASCTCLPTATSLKSSLVC
